# Supplementary material for: Does productive safety net program enhance livelihoods? Insights from vulnerable households in Wolaita zone, Ethiopia
Source: PLoS One. 2024 Apr 4;19(4):e0297780. doi: 10.1371/journal.pone.0297780 (PMC10994340; doi:10.1371/journal.pone.0297780)
Supplement: S1 File — (DOC) [file pone.0297780.s001.doc]

**Household survey questionnaire (HSQ)**

**Introduction and consent**

The purpose of this HSQ is to explore households’ vulnerability situations to the changing climate and investigate resilience capacities from adaptive, absorptive and transformative capacities perspectives in Wolaita *Zone*, Southern Ethiopia. This is therefore to collect detailed information on households’ vulnerability situations and explore the existing resilience capacities in the face of the changing climate. You are selected randomly for this survey and your participation is based on your willingness to take part. Therefore, your kind cooperation with honest responses to the HSQ will be vital for the overall success of the study. It is purely for academic purpose; results and any other details will not be used for un-intended purpose. To this end, anonymity and confidentiality of our respondents are highly guaranteed unless and otherwise under mutual agreement between the concerned parties.

**General instruction for the interviewer/enumerator**

- Please put the code chosen by the respondent in the space provided for some of the close-ended questions and encircle the chosen reposes for those which do not have space to put the codes;
- The respondent can choose more than one response for some questions (multiple response items);
- Skip the code to which it does not require the response;
- Write down the response for open-ended questions whenever the respondent provides and necessary;
- Respondent agrees to be interviewed Respondent does not agree to be interviewed

**...................................................1 .................................................. 2 → END**

**↓**

**MODULE 1: H**OUSEHOLD IDENTIFICATION

| **N** | **Date of the enumeration:** | **Time: Start: Finish: _** |
| --- | --- | --- |
|  | HH identification number: |  |
|  | *Wereda*: 1=Damote Gale; 2=Sodo Zuria 3=Duguna Fango | |
|  | *Kebele*: 1=Aro Wegera; 2=Konassa Bullasa; 3=Wondra Bolosso; 4=Humbo Larena; 5=Zalla Shasha; 6=Wereza Lasho; 7=Haba Gerera; 8=Kokate Maria Chare; 9=Dendo Werkicho; 10=Arso Woyde; 12=Duguna Damote Shinka | |
|  | Village/Gotte_______________________________________________________________ | |
|  | Agro-ecology type: 1=Highland 2= Midland 3=Lowland | |
| **Enumerator name: ______________** | | **Signature: _____________** |

MODULE 2: BASIC DEMOGRAPHIC INFORMATION

| Relation to the head [**code 1**] | Age in years (write years) | Sex [**code 2**] | Marital status  ***(****if age above 18*)  *[***code 3***]* | Religious affiliation [ **code 4**] | 1. Occupation   **[See code 5]** | | Highest level of education (completed) [for education level different from 1- 12  *[***code 6***]* | Length of years in the current residence [write *years]* | Responsibility in the community *[***code 7***]* |
| --- | --- | --- | --- | --- | --- | --- | --- | --- | --- |
|  |  |  |  |  | Main | Secondary |  |  |  |
|  |  |  |  |  |  |  |  |  |  |
|  |  |  |  |  |  |  |  |  |  |
|  |  |  |  |  |  |  |  |  |  |
|  |  |  |  |  |  |  |  |  |  |
|  |  |  |  |  |  |  |  |  |  |
|  |  |  |  |  |  |  |  |  |  |
|  |  |  |  |  |  |  |  |  |  |
|  |  |  |  |  |  |  |  |  |  |
|  |  |  |  |  |  |  |  |  |  |

**Codes for 1:**1= Head 2= Wife/Husband/Partner3= Son/daughter 4= Grandchild 5= Father/Mother 6= Sister/Brother 7= Niece/nephew 8= Uncle/Aunt 9=Son/Daughter-in-law 10= Father/Mother-in-law 11= Brother/Sister-in-law 12= Grandparent 13= Other relative of head or of his/her spouses 14=Servant (farm worker, herder, maid) 15= other (specify) _____________

**Codes for** **2**: 1=Male 2=Female

**Codes for 3:** 1=Married, single spouse; 2= Married with more than one spouse/ polygamous; 3=Single; 4=Divorced; 5=Widowed; 6= Separated

**Codes for 4**: 1=Orthodox; 2=Muslim; 3=Catholic; 4=Protestant, 5=Indigenous religions; 6= other (specify) _____________

**Cods for 5***:* 1=None, 2=Farmer; 3=Farm assistant, 4= Salaried work; 5=Student; 6= Farming/crop production and sales; 7= Livestock production and sales; 8=Non-farm product trader; 9=Beverage (*tella, tej, areke*, etc), 10= Wage labor (local); 11=Pensioner; 12=Handicraft, 13=Mining, 14=Carpentry, 15=House help, 16= Sale of wild/bush products (including charcoal;17=blacksmith 18=looking for work 19=other (specify) _____________

**Codes for 6**: 0=Cannot read and write; 13=10+1; 14=10+2; 15=10+3; 16=Certificate; 17=Diploma; 18=Degree; 19=Masters and above; 20=Informal education (can read and write); 21=KG (Completed)

**Codes for 7:** 1= None; 2**=**Religious leader; 3= Coordinator of community development work; 4=*Kebele* Administrator; 5= other (specify) _____________

1. Is this your village of origin? 1=Yes 2=No
2. If your place of birth is different from the current place, what is the reason for coming here? (**Multiple response is possible)**

1=Marriage; 2=Join relative; 3=Displacement by drought; 4=Displacement by flooding; 5=Divorce 6=Search for agricultural land; 7=War/conflict; 8=others specify____________________

1. Ethnicity of the household head: 1=Wolaita; 2 Gamo; 3=Dawro; 4=Sidama; 5=Amhara 6=Oromo 7=Tigray; 8=other (specify) _____________
2. Number of permanent household members: Male_________female_____________
3. Capable to work 1=Yes; 2=No
4. Reasons for not working or being incapable: 1=Disability; 2=Old age/aged; 3=Other health problem; 4=Others specify_________________
5. If agriculture is your major livelihood activity, for how long did you work on agriculture? (write in years) _________________________
6. Which farming system are you following currently? 1=Only crop production; 2= Livestock raring; 3=Mixed farming (Crop production and livestock raring)
7. Have you changed your farming system in the past 5-10 years? 1=Yes 2=No
8. If yes for QN # 2.18, why did you change the farming system you were following? (Multiple response is possible)

**1**=Decrease in rainfall amount; 2= Drought; 3=Decrease in productivity of livestock; 4=Decrease in productivity of land; 5=Decrease in grazing land; 6=increase in pest and disease; 7=Others specify_________________

**MODULE 3: HOUSEHOLD SOURCES OF INCOME (ON-FARM, OFF-FARM AND NON- FARM ACTIVITIES).**

1. What are the sources of livelihoods and estimated earnings for all members in the household? (Answer all that apply)?

| **Farming activities** | **Codes**  **(**1=Yes;2=No**)** | Number of months participated | Estimated yearly income from each |
| --- | --- | --- | --- |
| Crop production |  |  |  |
| Livestock rearing |  |  |  |
| Fruit production (Apple, mango, Banana) |  |  |  |
| Bee keeping |  |  |  |
| Off-farm activities |  |  |  |
| Non-farm activities |  |  |  |
| Other (specify) |  |  |  |

1. Do any of your household members work in activities apart from crop production? 1=Yes; 2=No
2. If yes for QN #3.2, what are the types of **off-farm** livelihood activities that you are engaged in the last 12 months?

| **No.** | **Type of off-farm activities** | **Participation code**  **(**1=Yes;2=No**)** | **Number of months engaged in off-farm activities** | **Estimated annual income earned in *Birr*** |
| --- | --- | --- | --- | --- |
|  | Sale of agricultural labor |  |  |  |
|  | Sharecropping (cash or food) |  |  |  |
|  | Livestock herding |  |  |  |
|  | Sale of fire wood or charcoal |  |  |  |
|  | Sale of grass or fodder |  |  |  |
|  | Sale of wood |  |  |  |
|  | Petty trading (salt, soap, sugar, etc.) |  |  |  |
|  | Migratory labor (for a week or more) |  |  |  |
|  | Remittances |  |  |  |
|  | Gifts/inheritance |  |  |  |

1. If yes for QN #3.2, in which of the **non-farm** activities that you are engaged in the last 12 months?

| **No.** | **Type of non-farm activities** | **QN# 3.4.1**  **Participation code**  **(**1=Yes;2=No**)** | **QN# 3.4.1.1**  **Number of months engaged in non-farm activities** | **QN# 3.4.2**  **Estimated annual income earned in *Birr*** |
| --- | --- | --- | --- | --- |
|  | Trading grains and pulses |  |  |  |
|  | Trading livestock |  |  |  |
|  | Drinks production and sales |  |  |  |
|  | Weaving /spinning |  |  |  |
|  | Carpentry |  |  |  |
|  | Pottery |  |  |  |
|  | Blacksmithing or metal work |  |  |  |
|  | Traditional healers | ` |  |  |
|  | Renting out pack animals |  |  |  |
|  | Others (specify) |  |  |  |

1. For what purpose, did you use the income obtained from non-farm/off-farm activities? (Multiple response is possible) 1=Buy food; 2=Saving; 3=Buy clothes; 4=Pay taxes; 5=Pay loan; 6=Buy agricultural inputs; 7=others specify____________________
2. If you think that there is a challenge to engage in non-farm activities, what do you think are the possible reasons? (Multiple response is possible) [**use tick√ mark in front of the selection**]

| 1. Lack of spare time from agriculture |  | 1. No employment opportunities |  |
| --- | --- | --- | --- |
| 1. Lack of awareness about its use |  | 1. Jobs are too far away |  |
| 1. Lack of work skills |  | 1. Poverty/lack of funds |  |
| 1. Unable to work due to old age |  | 1. Income is intermittent |  |
| 1. Health problem |  | 10. Others specify |  |

1. Is anyone from the family member work outside the community? 1= Yes; 2=No
2. If yes for QN # 3.7, how many in number? Males___________Females_________

# MODULE 4: HOUSEHOLD EXPENDITURE AND CONSUMPTION INDICATORS

# We would like to ask you about all the food that was bought for consumption or was consumed from your own (beteseb's) stock, IN THE LAST WEEK.

# In last week, did your household consume any of the following? (FOOD-ITEM EXPENDITURE)

| **Food type** | 1. How much was purchased? How much was spent? | | | 2. Did you consume this food from your own harvest or your own stock? How much? | | 3. Did you receive this food as a GIFT, a LOAN, as WAGE IN KIND or as BARTER? How much? Who gave you this food? GIVE AMOUNT CONSUMED IN THE LAST WEEK | | |
| --- | --- | --- | --- | --- | --- | --- | --- | --- |
|  | **Response**  **1=Yes 2=No** | **Amount (code 1)** | **Total expenditure** | **Response**  **1=Yes 2=No** | **Amount (code 1)** | **Response**  **1=Yes 2=No** | **Amount (code1)** | **Source (code 2)** |
| Teff |  |  |  |  |  |  |  |  |
| Barley (Gebis) |  |  |  |  |  |  |  |  |
| Wheat(Sinde) |  |  |  |  |  |  |  |  |
| Maize (Bekolo) |  |  |  |  |  |  |  |  |
| Sorghum (Mashila) |  |  |  |  |  |  |  |  |
| Lentils (Misir) |  |  |  |  |  |  |  |  |
| Horse Beans (Bakela) |  |  |  |  |  |  |  |  |
| Cow Peas (Ater) |  |  |  |  |  |  |  |  |
| Chick Peas(Shimbra) |  |  |  |  |  |  |  |  |
| Milk/yoghourt (ergo) |  |  |  |  |  |  |  |  |
| Mutton (yebeg)/goat meat (yefiyel siga) |  |  |  |  |  |  |  |  |
| Chicken |  |  |  |  |  |  |  |  |
| Eggs |  |  |  |  |  |  |  |  |
| Butter/cheese |  |  |  |  |  |  |  |  |
| Tella/Tej |  |  |  |  |  |  |  |  |
| Birra (Bottled) |  |  |  |  |  |  |  |  |
| Araqi/Kathikala |  |  |  |  |  |  |  |  |
| Potatoes |  |  |  |  |  |  |  |  |
| Sweet Potatoes |  |  |  |  |  |  |  |  |
| Green leaf vegetables |  |  |  |  |  |  |  |  |
| Enset |  |  |  |  |  |  |  |  |
| Others (code 3) |  |  |  |  |  |  |  |  |

**Codes for 1**: 1=Kilograms; 2=Quintal; 3=Chinet; 4=Dawula; 5=Kuna; 6=Silicha; 7=Esir; 8= 9=Litter; 10=Kil; 11= Gan;13=Ensira;14=Tassa; 15=Big Madaberia; 16=Small Madaberia; 17=Shekim 18=Others specify____________

**Codes for 2:** 1=Family, local; 2=Family, non-local; 3=Neighbour/village member; 4= Individual from outside village; 5= Gift from government; 6= Gift from aid agency, NGO; 7= Food-for-work (PSNP); 8=Wages in kind; 9=Barter; 10=Loan; 11=Other specify _____________

**Codes for 3**: 1=Salt; 2 =sugar=cooking oil, 3= Spices/Karia/Berbere, 4= Bread (Dabo), 5= Macaroni/Spaghetti, 6= Honey 7= coffee 8=soft drinks and others specify____________

# Has the household purchased any prepared foods, or paid to eat food outside the household in the last week? (1 =Yes 2=No (If no, skip to QN#4.4.)

# What was the total expenditure on prepared foods and food eaten outside the household in the last week? ___________(Birr)

# NON-FOOD ITEM EXPENDITURE AND HOUSEHOLD CONSUMABLES: PART I

1. Did the household purchase any of the following for its own consumption during the last MONTH? If so, where did you purchase these?

| **Commodity** | **Total expenditure (Birr)** | **The place purchased (code)** |
| --- | --- | --- |
| Matches/Batteries |  |  |
| Candles (tua'af), incense |  |  |
| Laundry soap/OMO/endod/besana leaves, Hand soap |  |  |
| Other personal care goods (incl. sendel, matent,) |  |  |
| Charcoal/ Firewood |  |  |

**Codes for 1:** 1=This village, 2=Another village, 3=Local market town/Sodo, 4=Regional center/Hawassa, 5=Addis Ababa 6=Other (Specify)

**NON-FOOD ITEM EXPENDITURE: PART II**

1. IN THE LAST FOUR MONTHS, has the household purchased any of the following non-food items?

| **Commodity** | **Total expenditure in the last four** | **Where purchased? (code 1)** |
| --- | --- | --- |
| Clothes/shoes/fabric for men, women, goys, and girls |  |  |
| Kitchen equipment (cooking pots, etc.) |  |  |
| Linens (sheets, towels, blankets) |  |  |
| Furniture |  |  |
| Building materials |  |  |
| Transport |  |  |
| Ceremonial expenses (weeding, holyday) |  |  |
| Contributions to *Iddir* |  |
| Donations to the church |  |
| Compensation and penalty |  |
| Voluntary contributions |  |
| Involuntary contribution (forced) |  |
| Other goods purchased (code 2) |  |  |

**Codes for 1:** 1=This village; 2=Another village; 3=Local market town/Sodo; 4=Regional center/Hawassa; 5=Addis Ababa; 6=Other (Specify)___________

**Codes for 2:** 1= Savings and credit scheme; 2= Repair and maintenance; 3=Cosmetics (Hair oil, butter, perfume); 4= Bicycle or motor bicycle; 5=Bio-Gas tube (Oxygen gas); 6= Labor cost/salary; 7=Jeba, Gembo, Mitad, Broom and other such items; 8=Payment to broker

**NON-FOOD ITEM EXPENDITURE: PART III**

1. IN THE LAST FOUR MONTHS, has the household purchased any of the following non-food items?

| **Commodity** | **Total expenditure in the last four** | **Where purchased? (code 1)** |
| --- | --- | --- |
| Modern medical treatment and medicines |  |  |
| Traditional medicine and healers |  |  |
| School fees |  |  |
| Other educational expenses (exercise books, pens, pencils, uniforms, maintenance, club fees) |  |  |
| Cigarettes, tobacco, suret, gaya |  |  |
| Alcoholic beverages |  |  |
| Others specify |  |  |

**Code 1:** 1=This village, 2=Another village, 3=Local market town/Sodo, 4=Regional center/Hawassa, 5=Addis Ababa 6=Other (Specify)

# MODULE 5: FOOD SECURITY INDICATORS

1. Indicate the months of not enough food, enough food and surplus food you have experienced during the last 12 months?

| 0=Not enough food/food insecure  1= Just enough food/food sufficient  2= Plenty of food/surplus or food secure | | | | | | | | | | | |
| --- | --- | --- | --- | --- | --- | --- | --- | --- | --- | --- | --- |
| **2008/2016** | | | | | | | | | | | |
| **Jan** | **Feb** | **Mar** | **Apr** | **May** | **June** | **July** | **Aug** | **Sept** | **Oct** | **Nov** | **Dec** |
|  |  |  |  |  |  |  |  |  |  |  |  |
| Total # of food needs met (all months with the score of 1 or 2) | | | | | | | | | | |  |
| Total # of food gaps (all months with the score of 0) | | | | | | | | | | |  |

1. Does the income you earn from non-farm or off-farm activities enable you to buy food for bridging the deficiency? 1=Yes 2=No
2. Did you or your household members face any food shortage in the last 12 months? 1= Yes; 2=No
3. If your household has faced food shortage over the last 12 months, what is the possible cause for this? (multiple response is possible)

1=Heavy rains/hailstorm; 2=Drought; 3=Diseases and pests; 4=Depleted soil nutrients; 5=Small land holdings; 6=Lack of enough money; 7=Traditional farming tools/system; 8=Big family size; 9=Other (Specify) ________

1. How did your household cope up during food shortage months?

1=Purchase of grain from market; 2=Food aid; 3=Food/Cash from work; 4=Support from friends; 5=Credit cash; 6=income from off-farm; 7=Income from non-farm; 8 =Sale animals and animal products; 9=Others (specify) ___________________

1. How much quintal is the actual annual grain requirement of your household? (Estimate) _________________ Quintals

**Food Access: Household dietary diversity score (HDDS) (Applicable to all households)**

| **No.** | HDDS QUESTIONS | **Response codes** | If yesterday was special or unusual day, skip this table |
| --- | --- | --- | --- |
| Now I would like to ask you about the types of foods that you or anyone else in your household ate yesterday during the day and at night. This does not include food that you ate at a restaurant or outside of the home.  Read the list of foods. Choose “yes” if anyone in the household ate the food in question. Choose “no” if no one in the household ate the food.  THE FOODS LISTED SHOULD BE THOSE PREPARED IN THE HOUSEHOLD AND EATEN IN THE HOUSEHOLD OR TAKEN ELSEWHERE TO EAT. DO NOT INCLUDE FOODS CONSUMED OUTSIDE THE HOME THAT WERE PREPARED ELSEWHERE. | |
|  |  |
|  | Any enjera, bread, rice, biscuits, or other foods made from teff, millet, sorghum, maize, rice, pasta, macaroni, wheat or barley or other cereal. | 1 = Yes, 2 = No |  |
|  | Any potatoes, yams, cassava, or any other foods made from roots or tubers? | 1 = Yes, 2 = No |  |
|  | Any vegetables? | 1 = Yes, 2 = No |  |
|  | Any fruits? | 1 = Yes, 2 = No |  |
|  | Any meet, beef, lamb, goat, wild game, chicken, liver, kidney, heart, or other organ meats? | 1 = Yes, 2 = No |  |
|  | Any eggs? | 1 = Yes, 2 = No |  |
|  | Any fresh or dried fish? | 1 = Yes, 2 = No |  |
|  | Any foods made from beans, peas, lentils, haricot beans, or nuts? | 1 = Yes, 2 = No |  |
|  | Any cheese, yogurt, milk, or other milk products? | 1 = Yes, 2 = No |  |
|  | Any foods made with oil, fat, or butter? | 1 = Yes, 2 = No |  |
| **Sum of categories above (a scale of 0-10)** | | |  |
|  | Any sugar or honey? | 1 = Yes, 2 = No |  |
|  | Any other foods, such as condiments, coffee or tea? | 1= Yes, 2 = No |  |

1. Did you participate in safety net programs in the past? 1= Yes; 2=No
2. Currently, are you a member of productive safety net programs? 1= Yes; 2=No

**MODULE 6: LIVELIHOOD VULNERABILITY AND RESILIENCE CAPACITY INDICATORS**

# Natural capital [Land, irrigated area and access to publicly owned resources]

# Do you have access to land for agricultural use? 1=Yes; 2= No

# If yes for QN #6.1, how did you get it?

1=Through land redistribution 2=Shared with the family/relatives 3=Inherited from parents 4=Share cropped- in 5=Rented from relatives 6=Purchased 7=Other (specify) _______________________

1. Do you have land use right/ownership certificate? 1= Yes 2=No
2. What are the total sizes of the following land types that you use?

| **Land type** | **Unit in local measure (kada or timad*)*** | **In hectare** |
| --- | --- | --- |
| Cultivated land |  |  |
| Fallow land |  |  |
| Grass and woodland |  |  |
| Forest land/wood lot |  |  |
| Homestead/backyard |  |  |
| Irrigated land |  |  |
| Total land holding in hectare |  |  |

1. What type of soil is your cultivated land? 1=Black 2=Brown 3=Red; 4= Other (specify) ___________
2. How did you plough your land? 1=Using pair of oxen/horses; 2=Using hand hoe; 3=Using machine or tractor; 4=Others specify____________________________
3. **List the total area of land operated in 2008/2009 EC production season?**

| **Land type** | **Area in hectare (ha)** | **Fertility of soil [see code 1]** | **Status of farming land [see code 2]** | **Slope of the land [see code 3]** |
| --- | --- | --- | --- | --- |
| Own land |  |  |  |  |
| Rented in land |  |  |  |  |
| Rented out land |  |  |  |  |
| Share crop in |  |  |  |  |
| Share crop out |  |  |  |  |
| Fallow land |  |  |  |  |

**Codes for 1**: 1=Not fertile; 2= Somewhat fertile 3=Fertile; 4=Highly fertile

**Codes for 2:** 1=Increased; 2=remain the same/no change; 3=decreased; 4=Do not know

**Codes for 3:** 1=Flat; 2=Somewhat hilly;3=Highly steep;4=Mountainous;5=Other specify____________

1. Have you sharecropped out your plot to other farmers on equal basis? 1=Yes 2=No
2. If yes for QN # 6.8, why did you sharecrop out? (Multiple response is possible):

1=Lack of draft power; 2=Lack of seed; 3=Unable to purchase technological inputs; 4=Elderly and unable to operate it; 5=Illness; 6=Having extra land; 7=Others, specify ________

1. Indicate your access to other publicly owned land resources

| **Indicator questions** | **Code definition** | **Response** |
| --- | --- | --- |
| 1. Do you have access to open or publicly owned grazing land? | 1=Yes 2= No |  |
| 1. Do you have access to open or publicly owned water source for livestock? | 1=Yes 2= No |  |
| 1. Do you have access to get firewood from open or publicly owned forested land? | 1=Yes 2= No |  |

1. What benefit do you get from the open or publicly owned land?

1=Grazing livestock; 2=Collecting fire wood; 3= Fire wood for selling; 4=Source of construction materials; 5=Other (specify) _____________________

1. What are the main constraints to your farmlands?

1= Erosion; 2= Waterlogging; 3= Poor soil fertility; 4=Susceptibility to frost; 5= High concentration of stones on the topsoil; 6= High concentration of stones on the topsoil; 7= Salinity; 8= Highly sandy; 9=Water scarcity-inaccessibility to water or drought; 10=Other (specify) _____________________

1. Did farm land related challenges force you to enhance the use of soil and water conservation management techniques (terracing, agroforestry, etc.)? 1=Yes 2=No
2. If yes for QN # 6.13, what is the size of your land area covered with improved soil and water conservation practices in hectare? _____________
3. Which measure(s) do you practice to minimize soil erosion on your own farm, and in your community at large? (Multiple response is possible)

1= Terracing; 2= Crop rotation; 3= Using composite; 4= Tree planting; 5= Soil or stone bunds; 6=Contour ploughing; 7=Furrowing; 8= Strip cultivation; 9= Other (specify) _____________________

1. Which of the following land management practice do you carry out in order to maintain and replenish the soil fertility of your farmlands? (Multiple response is possible)

1= Fallowing (field rotation); 2= Crop rotation; 3= Manuring; 4= Use of fertilizers (e.g., Dap, Urea, and blended fertilizer); 5= Inter-cropping; 6= Other (specify) _____________________

1. How do you see the status of your land size over the last 5-10 years?

1= Highly increased; 2= Increased; 3= No change; 4= Decreased; 5= Highly decreased

1. If your answer is decreasing for QN # 6.17, what is the possible reason for the decrease for farm land size? (Multiple response is possible)

1=increased demand for agricultural land; 2=land degradation; 3=land fragmentation; 4=conversation of farm land to non-farm activities; 5=limited carrying capacity or population pressure; 6=others specify________

1. **Crop Production**
2. Would please indicate the type of crops/vegetables you have produced/, land area covered by crops, amount sold, and income earned from sales of crops during the year 2008/2009 E.C

| Crop types | Have you produced […]?  1=Yes; 2=No | Area covered  (Timad)  (Kada) | Production (qt) (quintal/hectare) | Amount sold (quintals) | Income from sales (Birr) |
| --- | --- | --- | --- | --- | --- |
| Maize |  |  |  |  |  |
| Teff |  |  |  |  |  |
| Wheat |  |  |  |  |  |
| Barley |  |  |  |  |  |
| Haricot bean |  |  |  |  |  |
| Sorghum |  |  |  |  |  |
| Lentil |  |  |  |  |  |
| Coffee |  |  |  |  |  |
| Tomato |  |  |  |  |  |
| Potato |  |  |  |  |  |
| Cassava |  |  |  |  |  |
| Taro |  |  |  |  |  |
| Onion |  |  |  |  |  |
| Enset |  |  |  |  |  |
| Carrot |  |  |  |  |  |
| Beet root |  |  |  |  |  |
| Other specify |  |  |  |  |  |

1. What are criteria used to select crops for production? (Multiple response is possible)

1=Drought tolerance; 2=Pest and disease tolerance; 3=The time it takes to mature; 4= High market value; 5=High yield crop/productivity; 6=others, specify **_________**

1. Would you list the type of agricultural inputs you used in the 2008/2009 cropping year?

| **Type of agricultural inputs** | | **Responses**  **1=Yes 2=No** | **Total amount used in Kg.** | **Total amount of costs incurred** | **Total area covered using inputs (in Ha)** |
| --- | --- | --- | --- | --- | --- |
| Chemical fertilizers | DAP |  |  |  |  |
|  | Urea |  |  |  |  |
|  | Blended fertilizer |  |  |  |  |
| Pesticides/herbicides | |  |  |  |  |
| Improved seeds | |  |  |  |  |
| Others specify | |  |  |  |  |

1. If there are constraints in the use of agricultural inputs, what are the problems? (Multiple response is possible) 1=Drought/erratic rainfall; 2=High price of inputs; 3=Lack of cash; 4=Indebtedness; 5 =Farm land is inappropriate to use of fertilizers; 6=Crop disease; Excessive rain/flooding; 8=Unavailability of improved seed; 9=Untimely input distribution; 10= Other, please specify______________________
2. In general, what is the trend of your crop production for the following crop types over the last 5-10 years?

| **Crops produced** | **Trends in crop production** | | | | |
| --- | --- | --- | --- | --- | --- |
|  | **Highly decreased**  **(1)** | **Decreased**  **(2)** | **No change**  **(3)** | **Increased**  **(4)** | **Highly increased**  **(5)** |
| 1. Maize |  |  |  |  |  |
| 2. Wheat |  |  |  |  |  |
| 3. Barely |  |  |  |  |  |
| 4. Teff |  |  |  |  |  |
| 5. Sorghum/ |  |  |  |  |  |
| 6. Peanuts |  |  |  |  |  |
| 7. Chickpea (shinbira) |  |  |  |  |  |
| 8. Bean (baqella) |  |  |  |  |  |
| 9. Pea (atar) |  |  |  |  |  |

1. What are the possible reasons for any increase in your cultivated land productivity? (Multiple response is possible)

1=Increased soil fertility; 2= Improved seed supply; 3= Improved agrochemical use; 4= Improved use of organic fertilizer; 5=Suitable weather conditions/good rainfall; 6=Soil and water conservation practices; 7= Other, please specify______________________

1. What are the possible reasons for any decrease of your cultivated land productivity? (Multiple response is possible)

1=Land degradation; 2=Lack of timely input supply; 3= Lack of oxen; 4=Erratic rainfall /variability;5=Drought; 6= Land scarcity; 7= Non-use of fertilizer; 8=Pests and crop diseases; 9= Other, please specify______________________

1. **Livestock Ownership/Production**
2. Please indicate the number of livestock owned by the household and the estimated current value per each

| **Type** | **Number of livestock ownership status** | | **Equivalence in cash currently (in *birr*)** |
| --- | --- | --- | --- |
|  | Last year | This year |  |
| 1. Cows |  |  |  |
| 1. Oxen |  |  |  |
| 1. Bulls |  |  |  |
| 1. Heifers |  |  |  |
| 1. Calves |  |  |  |
| 1. Sheep |  |  |  |
| 1. Goats |  |  |  |
| 1. Mules |  |  |  |
| 1. Horses |  |  |  |
| 1. Donkeys |  |  |  |
| 1. Chicken |  |  |  |
| 1. Bee colony |  |  |  |

1. How do you perceive the current livestock production compared to 5-10 years ago?

1= Highly increased; 2= Increased; 3= No change; 4= Decreased; 5= Highly decreased

1. If decreased for QN #6.27, what would be the cause? (Multiple response is possible)

1=Livestock disease prevalence; 2=Shortage of grazing land; 3=Natural disaster (e.g. drought); 4=Scarcity of fodder; 5=Shortage of hybrid; 6= Lack of sufficient veterinary services; 7= Shortage of water; 8= others (specify) **___________________________**

1. **Household Assets Ownership**

# Could you please tell us the number of productive assets you own currently, number of each asset and the cash equivalence of each asset in Birr?

| **Type of assets** | **Response 1=Yes; 2=No** | **If yes for each, number of asset owned** | **Cash equivalence of the asset** |
| --- | --- | --- | --- |
| 1. Axe (Metarabia) |  |  |  |
| 1. Machete |  |  |  |
| 1. Sickle (machid) |  |  |  |
| 1. Spade (Akafa) |  |  |  |
| 1. Hoe(doma) |  |  |  |
| 1. Bucket |  |  |  |
| 1. Grain mill(weficho) |  |  |  |
| 1. Plough(maresha) |  |  |  |
| 1. Plow/Yoke/ Kenber |  |  |  |
| 1. Plow/ Beam/ |  |  |  |
| 1. Plow/Share |  |  |  |
| 1. Horse/Mule/Ox Cart |  |  |  |
| 1. Modern bee hive |  |  |  |
| 1. Traditional bee hive |  |  |  |
| 1. Weaving equipment |  |  |  |
| 1. Gotera or Dibignit |  |  | Not applicable |
| 1. Hammer (fas or martelo) |  |  |  |
| 1. Saw(megaz) |  |  |  |

# Could you please tell us the number of household assets you own currently, number of each asset and the cash equivalence of each asset in Birr?

| **Type of assets** | **Response**  **1=Yes 2=No** | **If yes, number of assets owned** | **Cash equivalence in Birr** |
| --- | --- | --- | --- |
| 1. Blankets/gabis |  |  |  |
| 1. Bed (alga) |  |  |  |
| 1. Chairs |  |  |  |
| 1. Tables |  |  |  |
| 1. Cupboard (Sanduk/Kumsatin) |  |  |  |
| 1. Leather Mat (Kurbet, Agoza, Debdab) |  |  |  |
| 1. Flashlight (torch) |  |  |  |
| 1. Watch/clocks |  |  |  |
| 1. Kerosene stove |  |  |  |
| 1. Radio/cassette player |  |  |  |
| 1. DVD player |  |  |  |
| 1. Television |  |  |  |
| 1. Mobile phone |  |  |  |
| 1. Bicycle |  |  |  |
| 1. Motor bicycle |  |  |  |
| 1. Iron (Kawuya) |  |  |  |
| 1. Leather Pouch (Silicha) |  |  |  |
| 1. Mechegia (Leather Sofa) |  |  |  |
| 1. Jewelry/Gold/Maria Theresa Coin, Ring |  |  |  |
| 1. Guns(Tebmenja) |  |  |  |
| 1. Spear/Sword (Gorade) |  |  |  |
| 1. Shield (Gasha) |  |  |  |

1. Has any household member lost any of the above mentioned productive assets due to the last year (2007/2008) drought? 1= Yes 2= No
2. If yes for QN # 6.31, what are the major impacts of the drought on your productivity that your household has experienced during the last 12 months? (Multiple response is possible)

1= Loss of productive assets; 2= Loss of household income; 3=Reduction in household consumption; 4= Asset and income loss; 5= Asset loss and reduced consumption; 6= Income loss and reduced consumption; 7= Other specify___

1. **Access to Financial Capital**
2. Did you have access to any cash credit service from any source during the last 12 months?

1= Yes 2= No

1. If yes for QN# 6.33, please indicate your access to credit services, amount and purposes of loan?

| **No.** | **Source of credit** | **Amount of credit received in *Birr*** | **For what purpose, did you receive the money? (use code 1)** |
| --- | --- | --- | --- |
|  | Marketing cooperative |  |  |
|  | Local money lender |  |  |
|  | Friends/relatives |  |  |
|  | NGOs |  |  |
|  | Omo MFIs |  |  |
|  | Banks |  |  |
|  | Vision fund |  |  |
|  | Religious institutions |  |  |
|  | Saving group |  |  |
|  | Women group |  |  |
|  | Iddir |  |  |
|  | Equib |  |  |
|  | Others, specify |  |  |

**Codes for 1**: 1= Petty trade; 2 = Food grain purchase; 3 = Input purchase for crop production; 4 = Animal purchase; 5 = Other income generating activities; 6 = Repay debt; 7= others, specify _____________

1. If no for QN # 6.36, what was the problem?

1= Did not want to take loan; 2=couldn’t find a loan that met my needs; 3=Afraid I couldn’t pay back; 4= No loan providers in the locality; 5= others, specify _____________

1. Do any of the household members have cash saving? 1=Yes 2=No
2. If yes for QN # 6.36, where is the saving held?

1= Credit or micro-finance group; 2=Banks; 3=Religious institutions; 4=Saving group; 5=Vision fund; 6=Women’s group; 7=Others specify_________________

1. What is the total amount of saving in *Birr*? _________________________
2. What is the primary purpose of saving?

1=To use in time of emergencies; 2=To buy livestock; 3=To buy agricultural inputs; 4=To pay debates; 5=Others specify________________

1. Do any of the household members have existing debate from any one? 1=Yes 2=No
2. If yes for QN # 6.40, what is the total amount of credit to be paid? __________________

# Network/Relationship (Social Capital)

| **No.** | **Indicator questions** | **Response code** |
| --- | --- | --- |
|  | In the past months in a year, did relatives or friends help you and your family (e.g., to get medical care or medicines, sell animal products or other goods produced by the household, take care of children, worked in field? | 1=Yes 2= No |
|  | In the past months in a year, did you and your family help relatives or friends? | 1=Yes 2= No |
|  | Has anyone from your family participate in informal association (*Iddir, equib, mahaber et*c) | 1=Yes 2= No |
|  | If yes for QN # 3, are there bylaws to govern the groups? | 1=Yes 2= No |
|  | Has anyone from your family participate in informal association (*debo or* working groups)? | 1=Yes 2= No |
|  | If yes for QN # 4, what is the number of non-working days/month in the debo? _______________days | |
|  | How do you rate the culture of working together in your community?  1=Very weak, 2=Weak, 3=Fair, 4=Good, 5=Very Good | |
|  | Has anyone of your family member or involve in formal association? (local leadership, one-five organization, & others? | 1=Yes 2= No |
|  | Has anyone of your family member or involve in cooperative promotion? | 1=Yes 2= No |
|  | Has anyone of your family member or involve in household asset building program/HABP? | 1=Yes 2= No |
|  | Has anyone of your family member or involve in disaster reduction and early warring/DRR-EW committee? | 1=Yes 2= No |
|  | Has anyone of your family member or involve in hazard insurance schemes? | 1=Yes 2= No |
|  | Has anyone of your family member or involve in shock preparedness and mitigation program or schemes? | 1=Yes 2= No |
|  | Has anyone of your family member or involve in vision fund (VF)? | 1=Yes 2= No |
|  | Has anyone of your family member or involve in women group? | 1=Yes 2= No |
|  | Has anyone of your family member or involve in religious group? | 1=Yes 2= No |
|  | Has anyone of your family member or involve in charitable group? | 1=Yes 2= No |

1. Health, Trainings /Skills

| **No.** | **Indicator questions** | **Response code** |
| --- | --- | --- |
|  | Is anybody in your family chronically ill? | 1=Yes 2= No |
|  | Has anyone in your family been so sick in the past 1 month that they had to miss work or school? | 1=Yes 2= No |
|  | Has anyone in your family been suffered by TB, Malaria, Cholera or other communicable diseases in the past six months? | 1=Yes 2= No |
|  | Has anyone in your household has died due to illness or natural disasters (drought, floods, landslides, malnutrition) in the past 5-10years? | 1=Yes 2= No |
|  | Has anyone in your household has been injured due to the climate related disasters (drought, flood, epidemics etc…) in the past 5-10years? | 1=Yes 2= No |
|  | Do you have access to health extension services in your locality? | 1=Yes 2= No |
|  | If yes for QN # 6, 1=Daily 2=Weekly 3=Fortnightly 3=Monthly 4=biannually 5=Annually | |
|  | Do you send your children to school? | 1=Yes 2= No |
|  | Have you received any training over the last 12 months? | 1=Yes 2= No |
|  | If your answer is yes for QN # 9, could you state the number of trainings you have attended over the last 12 ______________months? |  |
|  | Type of trainings attended (multiple response is possible)  1= Crop Management/Production; 2= Livestock Production (animal health/fattening); 3= Financial Literacy; 4= Health/Family Planning; 5= Climate change adaptation/conservation; 6= Business skills (income generating activities); 7= Value Addition (Marketing); 8= Nutrition (dietary diversity); 9= Poultry Production; 10= Early warning and disaster risk management; 11=Others specify____________________________ | |
|  | Do you have additional skills? | 1=Yes 2= No |
|  | If yes for QN # 11, what are these skills? | |
|  | 1______________________2____________________3________________ | |

# MODULE 7: ADDITIONAL RESILIENCE CAPACITY INDICATORS

1. **Physical capital**

| **No.** | **Indicator questions** | | **Response code** |
| --- | --- | --- | --- |
|  | Do you or your family own a house? | | 1=Yes 2= No |
|  | What is the construction materials used for outside walls and roof of your house?  1= Mud/dung (chika/ebet); 2=Wood; 3=Galvanised iron (korkoro); 4= Stone/Brick/Concrete/Cement; 5= Thatch (sar) 6=Bamboo 7=others specify | | |
|  | Do you have access to clean and safe [drinking] water supply? | | 1=Yes 2= No |
|  | What is the main drinking water for your household? 1=Piped in 2=Tube well/borehole with pump 3=Protected dug well 4=Open/unprotected well 5=Protected spring 6=River/stream | | |
|  | Who normally collect water for the household? 1=Men 2=Women 3=Both | | |
|  | Do you use a toilet [does not include outdoors defecation]? | 1=Yes 2= No | |
|  | What is the main source of lighting for the house? 1=Bottle lamp 2=Kerosene 3=Candle 4=Wood fire 5=Other (specify) | | |
|  | What is the main source of cooking fuel for the household? 1=Wood 2=Charcoal 3=Gas 4= Kerosene 5=Dung 6=Crop residue 7=Other (specify) | | |
|  | If you collect wood for QN #8, how long does it takes to go there and comeback? _________ min/hours | | |
|  | Who normally collect firewood for the family? 1=Men 2=Women 3=Both | | |

1. **Access to Infrastructure**

| **No.** | **Indicator questions** | **Walking distance in KM** | **Walking distance in minutes** |
| --- | --- | --- | --- |
|  | How far is your home from all weather-roads? |  |  |
|  | How far is your home from healthcare center? |  |  |
|  | How far is your home from the savings and credit institutions? |  |  |
|  | How far is your home from the village market/within the *Kebele*? |  |  |
|  | How far is your home from the main market (central for *Wereda*) |  |  |
|  | How far is your home from the primary school? |  |  |
|  | How far is your home from the secondary school? |  |  |
|  | How far is your home from the farmers training center? |  |  |
|  | How far is your home from the veterinary services? |  |  |
|  | How far is your house from the save drinking water? |  |  |

1. **Access to Information and Services**

| **No.** | **Indicator questions** | **Response code** |
| --- | --- | --- |
|  | Do you have access to climate related information? | 1=Yes 2= No |
|  |  |  |
|  | Do you have access to early warning information system? | 1=Yes 2= No |
|  | If yes for QN # 2, what type of early warning information providing structure are put in place? | 1=Formal/government; 2=Informal/traditional |
|  | If yes for QN # 2, how often have you been informed about the potential risk of natural disasters (erratic rain fall, flooding, land slid, outbreak of crop pests and diseases) | 1=Frequently; 2=Occasionally/seasonally; 3=Rarely |
|  | Who provides the information on early warning? | 1=Government; 2=NGOs; 3=CBOs; 4=All of them |
|  | How do you rate the contribution of the early warning system for you to withstand from the shocks? | 3=High; 2=Medium; 1=Low; 0=None |
|  | Do you have access to agricultural extension services? | 1= Yes 2= No |
|  | Does the presence of extension services help you to improve productivity and production? | 1= Yes 2= No |
|  | How often do you meet and get advice from the extension agent? 1=Daily; 2=Weekly; 3=Once in two weeks;4=Monthly; 5=Once in three months 6=None/not at all | |
|  | Do you get important information related to agriculture and market from this information?  center/source? | 1= Yes 2= No |
|  | Do you have access to irrigation sources (pond, diversion canal, etc.) | 1= Yes 2= No |
|  | Do you have access to security services that can reach the community within 1 hour? | 1= Yes 2= No |
|  | Do you have access to micro-insurance scheme or program in your locality? | 1= Yes 2= No |
|  | If yes for QN # 13, what type of micro-insurance program are you involved in? | 1=Crop 2=Livestock 3=Both 4=Others specify______________ |

1. Who provides the services in your locality? 1=Government; 2=NGOs**;** 3=CBOs; 4=Private business; 5=All of them
2. How do you rate the effectiveness of service delivery? 5**=** Most effective; 4= Effective; 3= Moderate; 2= Less effective; 1= Ineffective
3. What are your main early warning information needs? (multiple response is possible)

1=Information on rainfall; 2=Market information; 3=Updates on harvesting time; 4=Updates on growing time; 5=About impending possible hazard 6=Others (specify)____________________

1. Which mechanisms are being used to disseminate EW information? (multiple response is possible)

1= Radio and TV; 2= Printing media (newspaper, magazine, etc); 3= Community meetings; 4=One to one communication; 5=Through *Kebele* EW committees; 6=Through local Command post; 7=Agriculture Extension Officers; 8=NGOs; 9= Others (specify)______________________

1. How do you rate the effectiveness of information dissemination mechanisms? 5**=** Most effective; 4= Effective; 3= Moderate; 2= Less effective; 1= Ineffective
2. If it is ineffective for QN # 7.5, why?

1= Not timely disseminated information; 2=Less relevant information; 3=lack of services to act timely; 4=awareness raising and mobilization was not enough; 5=lack of support provided for timely action; 6=Others (specify)______________________

1. **Availabilit**y of Formal and Informal Support Mechanisms

| **No.** | **Indicator questions** | **Response code** |
| --- | --- | --- |
|  | Is there any institution in your locality where people can receive food assistance in time of shock or emergences? | 1= Yes 2= No |
|  | Is there any institution in your locality where people can receive non-food assistance in time of shock or emergences? | 1= Yes 2= No |
|  | Is there any institution in your locality where people can receive assistance due to losses of livestock? | 1= Yes 2= No |
|  | Is there any institution working on disaster response program from government or NGOs? | 1= Yes 2= No |

**MODULE 8:** FARMERS’ PERCEPTION TO CLIMATE VARIABILITY OR CHANGE

1. To what extent would you agree or disagree that the options indicated in the table below apply as possible reasons to responses by your household to the climate trend (Changes of temperature and precipitation)

| **No.** | **Perception indicators** | **Level of agreement or disagreement (five-point scale)** | | | | |
| --- | --- | --- | --- | --- | --- | --- |
| **Change in temperature** | | Strongly agree (5) | Agree (4) | Neutral (3) | Disagree (2) | Strongly disagree (1) |
|  | Increased temperature |  |  |  |  |  |
|  | No change temperature |  |  |  |  |  |
|  | Decreased temperature |  |  |  |  |  |
|  | Rainy season temperature decreased |  |  |  |  |  |
|  | Dry season temperature increased |  |  |  |  |  |
|  | Increase number of hot days |  |  |  |  |  |
|  | Number of cold days increased in years |  |  |  |  |  |
| **Change in Amount rainfall** | |  |  |  |  |  |
|  | Increased precipitation |  |  |  |  |  |
|  |  |  |  |  |  |  |
|  | No change in precipitation |  |  |  |  |  |
|  | Decreased precipitation |  |  |  |  |  |
|  |  |  |  |  |  |  |
|  | Late start of precipitation |  |  |  |  |  |
|  | Early cessation of precipitation |  |  |  |  |  |
|  | Belg rain decrease |  |  |  |  |  |
|  | Main rain season rain decrease |  |  |  |  |  |

**MODULE 9: CLIMATE INDUCED SHOCKS INDICATORS**

1. Have you ever faced crop failure during the last five to ten years?

1=Yes 2=No

1. If yes for QN# 9.1, what are the main reason for the crop failure? [Multiple response is possible]

1=Erratic rainfall; 2=Improved seed security and high price; 3=Unaffordable price of fertilizers; 4=Low level of soil fertility; 5=Pest and disease; 6=Shortage of farm oxen; 7=Other (specify)________________

1. Have you ever experienced flooding due to erratic rainfall over the last 10-20 years?

1=Yes 2=No

1. If yes for QN# 9.3, how do you rate the frequency of drought in your locality over the last 10-20 years?

5= Highly increased; 4= Increased; 3= No change; 2= Decreased; 1= Highly decreased

1. Have you ever experienced drought due to climate variability/change over the last 10-20 years?

1=Yes 2=No

1. If yes for QN # 9.5, how do you rate the frequency of flooding in your locality over the last 10-20 years?

5= Highly increased; 4= Increased; 3= No change; 2= Decreased; 1= Highly decreased

1. Have you ever experienced disease (crop, livestock and human) disease outbreak due to climate variability/change over the last 10-20 years?

1=Yes 2=No

1. If yes for QN# 9.7, how do you rate the frequency of disease outbreak in your locality over the last 10-20 years?

5= Highly increased; 4= Increased; 3= No change; 2= Decreased; 1= Highly decreased

1. **Self-Reported Climate Related Shock Indicators**
2. Please indicate your experiences with climate induced shocks, frequency of occurrences over the last 10-10 years and estimated costs of the damage due to the shocks

| **Experience of the following shocks** | **Frequency of shocks over the last (10-20 years)** | **Severity 1=High, 2=Medium, 3=low** | **Estimated costs of the damage on property/livelihood/health in *Birr*** |
| --- | --- | --- | --- |
| 1. Flooding |  |  |  |
| 1. Drought |  |  |  |
| 1. Crop failure |  |  |  |
| 1. Crop pests and diseases |  |  |  |
| 1. Livestock disease |  |  |  |
| 1. Infectious human disease/human health problem |  |  |  |
|  |  |  |  |

1. Who has been affected most by the above-mentioned shocks?

1=Men; 2=Women; 3=Children; 4=Elders; 5=All segment of the society

1. **Subjective Resilience Indicators**
2. **We would like to ask you about your level of agreement or disagreement if climate resulted shocks [INSERT THE SHOCK TYPE] were occurred/will be occurred, and your household would be/will be able to successfully deal with the threats posed in the past/to be posed in the future?**

| Type of shock anticipated [occurred in the past five years/last year] | Level of agreement on households’ capacity to withstand from shock [NAME THE SHOCK] in the past and level of agreement for each shock [PAST CAPACITY] (**code 1**) | Level of agreement for the likely occurrence of shocks [NAME THE SHOCK] in the future (next year/five years) and household capacity to withstand from each shock [FUTURE CAPACITY] (**code 1**) |
| --- | --- | --- |
| Flooding |  |  |
| Drought |  |  |
| Crop failure |  |  |
| Crop pests and diseases |  |  |
| Livestock disease |  |  |
| Infectious human disease |  |  |

**Codes for 1: 2=** Strongly disagree, 2= Disagree 3= Neutral, 4= Agree, 5= Strongly agree

1. Which of the following statements best describes the extent to which you and your household have been able to recover from the last drought (2015/6/2007/8 E.C)?

1=Did not recover; 2=Recovered some, but worse off than before drought; 3=Recovered to same level as before drought; 4=Recovered and better off; 5=Not affected by drought

1. Which of the following statements best describes your household’s ability to cope with and manage with future droughts or future periods of shocks or stress?

1=Unable to cope; 2=Able to cope, with changes income and food sources; 3=Able to cope without difficulty; 4=Others specify____________________________

**MODULE 10: PERCEIVED CLIMATE IMPACTS OF CLIAMTE CHANGE AND VARIABILITY**

1. Would you please indicate the type of impacts that climate change has brought to you or your household?

| **No.** | **Indicators questions** | **Dummy** | **Response** |
| --- | --- | --- | --- |
|  | Crop productivity decline | 1= Yes 2= No |  |
|  | Shortage of water for irrigation | 1= Yes 2= No |  |
|  | Shortage of water for home/animal consumption | 1= Yes 2= No |  |
|  | Emergence/resurgence of new pests (weeds and insects) | 1= Yes 2= No |  |
|  | Increased level of temperature | 1= Yes 2= No |  |
|  | Increased frequency of drought | 1= Yes 2= No |  |
|  | Increased frequency of flooding | 1= Yes 2= No |  |
|  | Geographic isolation/inaccessibility | 1= Yes 2= No |  |
|  | Livestock disease | 1= Yes 2= No |  |
|  | Crop pests and diseases | 1= Yes 2= No |  |
|  | Local conflict over diminishing resources | 1= Yes 2= No |  |
|  | Food price inflation | 1= Yes 2= No |  |

**MODULE 11: COPING OR ADAPTION STRATEGIES EMPLOYED**

1. Are there any adaptation strategies you have made for the change in climate (precipitation and temperature)? 1=Yes 2=No
2. If yes for QN #11.1, what adaptation strategies have you so far employed in response to long term change in precipitation and temperature over the last 5-10years?

| **No.** | **Key indicators or questions** | **Dummy** | **Response** |
| --- | --- | --- | --- |
|  | Planting different crops | 1= Yes 2= No |  |
|  | Shift to drought resistant crop varieties | 1= Yes 2= No |  |
|  | Practicing crop diversification | 1= Yes 2= No |  |
|  | Different/new planting dates | 1= Yes 2= No |  |
|  | Shortening length of growing period | 1= Yes 2= No |  |
|  | Move to different site | 1= Yes 2= No |  |
|  | Changes from crops to livestock | 1= Yes 2= No |  |
|  | Changes from livestock to crops | 1= Yes 2= No |  |
|  | Increasing water conservation on farms | 1= Yes 2= No |  |
|  | Use of irrigation (home gardens) | 1= Yes 2= No |  |
|  | Use of chemicals, fertilizers, manure and pesticides | 1= Yes 2= No |  |
|  | Shading and sheltering young plants | 1= Yes 2= No |  |
|  | Mixing crops and livestock (diversification) | 1= Yes 2= No |  |
|  | Adjusting livestock management practices | 1= Yes 2= No |  |
|  | Use of micro-insurance schemes | 1= Yes 2= No |  |
|  | Use of prayer and socio-cultural adaption schemes | 1= Yes 2= No |  |

**Thank you for your cooperation!!**
