# Supplementary figures and images for: Does productive safety net program enhance livelihoods? Insights from vulnerable households in Wolaita zone, Ethiopia
Source: PLoS One. 2024 Apr 4;19(4):e0297780. doi: 10.1371/journal.pone.0297780 (PMC10994340; doi:10.1371/journal.pone.0297780)

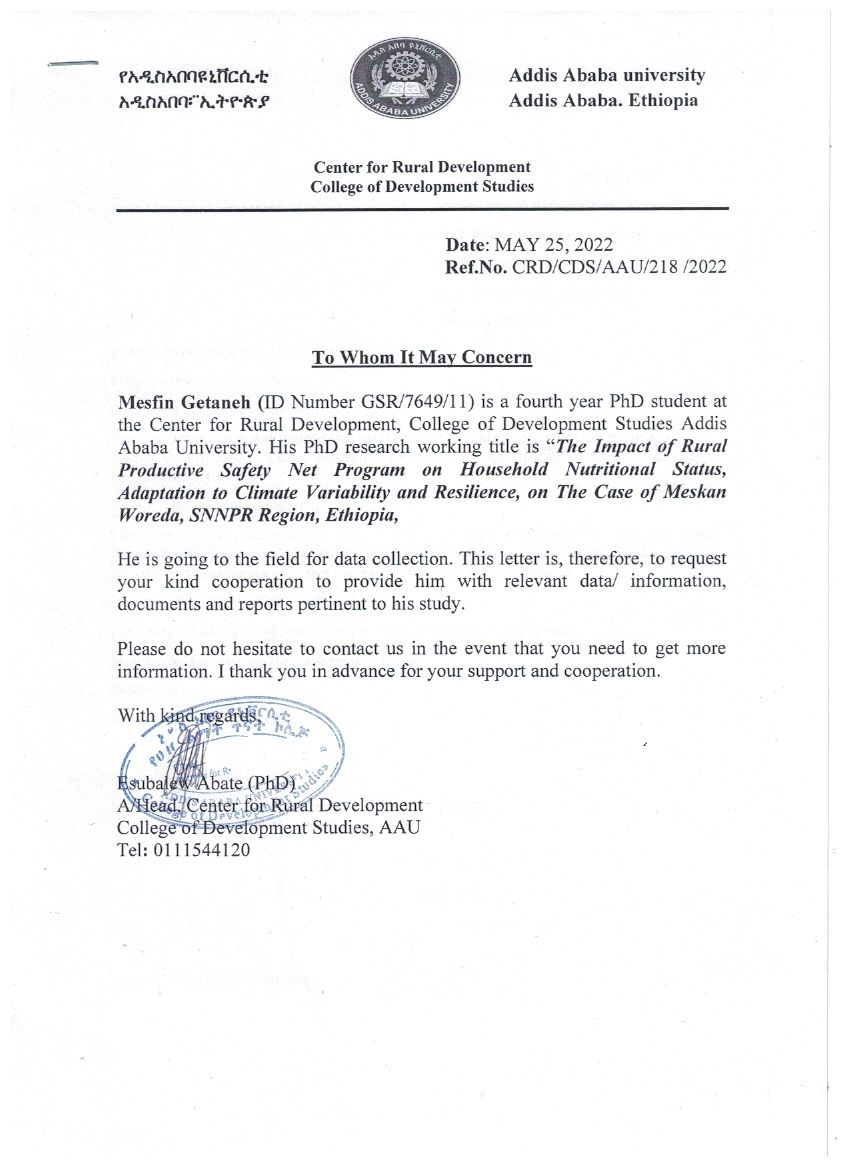

Supplement: S2 File — (JPG) [file pone.0297780.s002.jpg]
