# Supplementary material for: Does productive safety net program enhance livelihoods? Insights from vulnerable households in Wolaita zone, Ethiopia
Source: PLoS One. 2024 Apr 4;19(4):e0297780. doi: 10.1371/journal.pone.0297780 (PMC10994340; doi:10.1371/journal.pone.0297780)
Supplement: Attachment — Submitted filename: PONE-D-23-32019_Comments.pdf [file pone.0297780.s004.pdf]

PLOS ONE

Does Productive Safety Net Program Enhance Livelihoods? Insights from Vulnerable Households in Wolaita Zone, Ethiopia

--Manuscript Draft--

**Manuscript Number:**	PONE-D-23-32019
**Article Type:**	Research Article
**Full Title:**	Does Productive Safety Net Program Enhance Livelihoods? Insights from Vulnerable Households in Wolaita Zone, Ethiopia
**Short Title:**	Does Productive Safety Net Program Enhance Livelihoods?
**Corresponding Author:**	MESFIN WOLDEMICHAEL<br>Addis Ababa University<br>Addis Ababa, ETHIOPIA
**Keywords:**	livelihood; treatment effects model; productive safety net program; Impact; Ethiopia
**Abstract:**	Abstract<br><br>Background<br><br>The impact of Productive Safety Net Programs (PSNPs) on food security, poverty, and livelihoods has been examined in several studies. While some studies found positive impacts on food security and agricultural productivity, there are still gaps in understanding the long-term effects of these programs on poverty reduction and food security. This study aims to investigate the impact of PSNP on the livelihood of beneficiaries based on indicators including access to basic services, income, expenditure on food, adaptive capacity, and dietary diversity.<br><br>Methods<br>This article used treatment effects model taking into account access to basic services, income, food expenditure, assets, adaptive capacity, and household dietary diversity as outcome variables of interest. 300 respondents were randomly selected from the population of safety net beneficiaries and non-beneficiaries in the study area. Out of the 300 respondents, 150 were selected as the treatment group, who were beneficiaries of the safety net program. The algorithms used in analysis include regression adjustment, inverse probability weighing and propensity score matching.<br><br>Results<br>The study's results reveal that the PSNP has no statistically significant impact on access to basic services and income based on all three algorithms, yet it does show a significant and negative effect on food expenditure and assets. The coefficients across all three models consistently demonstrate this negative impact, underscoring its statistical significance at the conventional significance level (p= 0.05). This suggests that the PSNP leads to a reduction in food expenditure. Furthermore, the analysis highlights substantial differences between PSNP members and non-members across all three variables, with non-members having higher mean values for assets. This difference is statistically significant at the 1% level, suggesting that membership in the PSNP has a tangible impact on asset ownership. The study's mixed findings emphasize the complexities of designing effective social protection programs that can adequately address the multifaceted nature of poverty.
**Order of Authors:**	MESFIN WOLDEMICHAEL
	Bamlak Alamirew, PHD
**Additional Information:**	
**Question**	**Response**
**Financial Disclosure**	The author(s) received no specific funding for this work.

Powered by Editorial Manager® and ProduXion Manager® from Aries Systems Corporation

Enter a financial disclosure statement that describes the sources of funding for the work included in this submission. Review the submission guidelines for detailed requirements. View published research articles from *PLOS ONE* for specific examples.<br><br>This statement is required for submission and **will appear in the published article** if the submission is accepted. Please make sure it is accurate.<br><br>**Funded studies**<br>Enter a statement with the following details:<br>• Initials of the authors who received each award<br>• Grant numbers awarded to each author<br>• The full name of each funder<br>• URL of each funder website<br>• Did the sponsors or funders play any role in the study design, data collection and analysis, decision to publish, or preparation of the manuscript?<br><br>Did you receive funding for this work?	
**Competing Interests**<br><br>Use the instructions below to enter a competing interest statement for this submission. On behalf of all authors, disclose any competing interests that could be perceived to bias this work—acknowledging all financial support and any other relevant financial or non-financial competing interests.<br><br>This statement is required for submission and **will appear in the published article** if the submission is accepted. Please make sure it is accurate and that any funding sources listed in your Funding Information later in the submission form are also declared in your Financial Disclosure statement.<br><br>View published research articles from	The authors have declared that no competing interests exist.<br>I have read the journal's policy and the authors of this manuscript have the following competing interests:We conducted this research independently and solely for academic purposes. The views, opinions, and findings presented in this study are based solely on the analysis of the data collected and are not influenced by any external interests.<br>To ensure transparency and impartiality, no financial or other forms of support were received from any funding agencies, companies, or organizations for this research.<br>The research was conducted in accordance with ethical principles and guidelines, and any potential bias or conflicts of interest were consciously minimized throughout the research process.

Powered by Editorial Manager® and ProduXion Manager® from Aries Systems Corporation

PLOS ONE for specific examples.<br><br>**NO authors have competing interests**<br><br>Enter: *The authors have declared that no competing interests exist.*<br><br>**Authors with competing interests**<br><br>Enter competing interest details beginning with this statement:<br><br>*I have read the journal's policy and the authors of this manuscript have the following competing interests: [insert competing interests here]*<br><br>* typeset	
**Ethics Statement**<br><br>Enter an ethics statement for this submission. This statement is required if the study involved:<br><br>• Human participants<br>• Human specimens or tissue<br>• Vertebrate animals or cephalopods<br>• Vertebrate embryos or tissues<br>• Field research<br><br>Write "N/A" if the submission does not require an ethics statement.<br><br>General guidance is provided below. Consult the submission guidelines for detailed instructions. **Make sure that all information entered here is included in the Methods section of the manuscript.**	All participants in the study gave their informed Written consent, and they were informed of the study's purpose and objectives before agreeing to participate. The methods of data collection and tools were verified and approved by the Addis Ababa university as part of partial fulfillment of the PhD study. Consent was obtained from participants verbally in the local language, and participants had the right to refuse to participate in the study.<br><br>To ensure participants' privacy and confidentiality, all data collected from the participants was coded, and no identifying information was collected. The researcher did not collect any personal or identifying information. The study also ensured that there was no harm caused to the participants. They were provided with information about the study's purpose and objectives, and any potential risks or adverse effects were communicated to the participants. Participants were assured that they have the right to withdraw from the study at any time. Moreover this, the study ensured that the sample was selected randomly to ensure equal representation of the study population. All participants had an equal chance of being selected to participate in the study.<br><br>Overall, the research was conducted with the utmost care and attention, making sure that the participants were not subjected to any harm, deceit, or coercion. The participants were notified that the research results would not be used to harm the participants or others but will serve to provide insights into the effectiveness of the Productive Safety Net Program in Ethiopia.

**Format for specific study types**<br><br>**Human Subject Research (involving human participants and/or tissue)**<br>• Give the name of the institutional review board or ethics committee that approved the study<br>• Include the approval number and/or a statement indicating approval of this research<br>• Indicate the form of consent obtained (written/oral) or the reason that consent was not obtained (e.g. the data were analyzed anonymously)<br><br>**Animal Research (involving vertebrate animals, embryos or tissues)**<br>• Provide the name of the Institutional Animal Care and Use Committee (IACUC) or other relevant ethics board that reviewed the study protocol, and indicate whether they approved this research or granted a formal waiver of ethical approval<br>• Include an approval number if one was obtained<br>• If the study involved *non-human primates*, add *additional details* about animal welfare and steps taken to ameliorate suffering<br>• If anesthesia, euthanasia, or any kind of animal sacrifice is part of the study, include briefly which substances and/or methods were applied<br><br>**Field Research**<br><br>Include the following details if this study involves the collection of plant, animal, or other materials from a natural setting:<br>• Field permit number<br>• Name of the institution or relevant body that granted permission	
**Data Availability**<br><br>Authors are required to make all data underlying the findings described fully available, without restriction, and from the time of publication. PLOS allows rare exceptions to address legal and ethical concerns. See the PLOS Data Policy and FAQ for detailed information.	Yes - all data are fully available without restriction

A Data Availability Statement describing where the data can be found is required at submission. Your answers to this question constitute the Data Availability Statement and **will be published in the article**, if accepted.<br><br>**Important:** Stating 'data available on request from the author' is not sufficient. If your data are only available upon request, select 'No' for the first question and explain your exceptional situation in the text box.<br><br>Do the authors confirm that all data underlying the findings described in their manuscript are fully available without restriction?	
**Describe where the data may be found in full sentences. If you are copying our sample text, replace any instances of XXX with the appropriate details.**<br><br>• If the data are **held or will be held in a public repository**, include URLs, accession numbers or DOIs. If this information will only be available after acceptance, indicate this by ticking the box below. For example: *All XXX files are available from the XXX database (accession number(s) XXX, XXX.)*.<br>• If the data are all contained **within the manuscript and/or Supporting Information files**, enter the following: *All relevant data are within the manuscript and its Supporting Information files.*<br>• If neither of these applies but you are able to provide **details of access elsewhere**, with or without limitations, please do so. For example:<br><br>*Data cannot be shared publicly because of [XXX]. Data are available from the XXX Institutional Data Access / Ethics Committee (contact via XXX) for researchers who meet the criteria for access to confidential data.*<br><br>*The data underlying the results presented in the study are available from (include the name of the third party*	ll relevant data are within the manuscript and its Supporting Information files.

and contact information or URL).<br>• This text is appropriate if the data are owned by a third party and authors do not have permission to share the data.<br><br>* typeset	
Additional data availability information:	

Manuscript clean

Click here to access/download;Manuscript;PSNP article final 30th Sept clean.docx

Does Productive Safety Net Program Enhance Livelihoods? Insights from Vulnerable Households in Wolaita Zone, Ethiopia

Mesfin Getaneh Woldemichael[1] and Bamlaku Alamirew Alemu[2]

Contact : [1]Mesfin12005@yahoo.com (corresponding author)

[2] bamlakalamirew@gmail.com

Table of Contents

Abstract i

1. Background ~~of the Study~~ 1

2. Data and Methods 3

3. Ethical consideration 6

4. Results and Discussion 7

4.2.1 Impact of PSNP on Access to Basic Services 9

4.2.2 Impact of PSNP on Income 10

4.2.3 Impact of PSNP on Food expenditure 12

4.2.4 Impact of PSNP on Assets 13

4.2.5 Impact of PSNP of Adaptive Capacity 14

4.2.6 Impact of PSNP on Dietary Diversity 15

5. Conclusions and Policy Implications 16

5.1 Conclusions 16

5.2 Policy Implications 17

Abstract

Background

The impact of Productive Safety Net Programs (PSNPs) on food security, poverty, and livelihoods has been examined in several studies. While some studies found positive impacts on food security and agricultural productivity, there are still gaps in understanding the long-term effects of these programs on poverty reduction and food security. This study aims to investigate the impact of PSNP on the livelihood of beneficiaries based on indicators including access to basic services, income, expenditure on food, adaptive capacity, and dietary diversity.

Methods

This article used treatment effects model taking into account access to basic services, income, food expenditure, assets, adaptive capacity, and household dietary diversity as outcome variables of interest. 300 respondents were randomly selected from the population of safety net beneficiaries and non-beneficiaries in the study area. Out of the 300 respondents, 150 were selected as the treatment group, who were beneficiaries of the safety net program. The algorithms used in analysis include regression adjustment, inverse probability weighing and propensity score matching.

Results

The study's results reveal that the PSNP has no statistically significant impact on access to basic services and income based on all three algorithms, yet it does show a significant and negative effect on food expenditure and assets. The coefficients across all three models consistently demonstrate this negative impact, underscoring its statistical significance at the conventional significance level (p= 0.05). This suggests that the PSNP leads to a reduction in food expenditure. Furthermore, the analysis highlights substantial differences between PSNP members and non-members across all three variables, with non-members having higher mean values for assets. This difference is statistically significant at the 1% level, suggesting that membership in the PSNP has a tangible impact on asset ownership. The study's mixed findings emphasize the complexities of designing effective social protection programs that can adequately address the multifaceted nature of poverty.

Key words: Livelihood, treatment effects model, productive safety net program, impact, Ethiopia.

1. Background of the Study

Productive Safety Net Programs (PSNPs) have emerged as crucial social protection measures in numerous low-income countries, with Ethiopia being a prominent example. These initiatives are designed to mitigate poverty, food insecurity, and vulnerability by offering a combination of cash and food transfers, alongside public works opportunities, to households in dire need. While PSNPs are recognized for their vital support to vulnerable households, their impact on food security, poverty reduction, and livelihoods remains a subject of ongoing debate. To comprehend the potential impact of PSNPs on livelihoods, scholars have employed theoretical frameworks such as the sustainable livelihoods approach (SLA) and the asset-based approach (ABA). The SLA underscores the importance of building resilient livelihood systems capable of withstanding shocks and offering diverse income opportunities (Ellis, 2000; Devereux, 2006). Similarly, ABA emphasizes asset accumulation as a means to reduce poverty and enhance food security (Barrett & Carter, 2013; Devereux & Sabates-Wheeler, 2004). Furthermore, PSNPs are viewed as a form of social protection, aimed at reducing poverty and vulnerability by providing basic social security (World Bank, 2012). Nonetheless, the role of social protection in promoting economic development and poverty reduction remains a topic of contention, with concerns about dependency and work disincentives (Mkandawire, 2005; Standing, 2011).

Numerous studies have extensively investigated the impact of Productive Safety Net Programs (PSNPs) on food security, poverty, and livelihoods. These studies have collectively revealed a range of positive effects. For instance, Alderman and Yemtsov (2014) conducted a comprehensive review highlighting the positive outcomes of PSNPs, particularly in terms of bolstering food security and agricultural productivity. Similarly, Belete and Bayu (2023) demonstrated the beneficial impact of PSNPs in Ethiopia, specifically in improving the food security of female-headed farm households. Moreover, Devereux (2016) provided a conclusive review emphasizing the effectiveness of PSNPs in enhancing food security within the sub-Saharan African context. Furthermore, research by Sibhatu et al. (2015) identified improved food security outcomes among PSNP beneficiaries in Ethiopia, corroborated by the World Bank's impact evaluation in 2015, which confirmed positive effects on food consumption, asset accumulation, and child nutrition.

Additionally, Krishnan et al. (2021) highlighted the PSNP's role in reducing poverty among participating households in Ethiopia.

However, the impact of PSNPs on livelihoods is recognized as a more intricate matter. Hirvonen, Hoddinott, Minten, and Stifel (2017) conducted a review of conditional cash transfers, underscoring the variable impact on food consumption, dietary diversity, and poverty reduction and emphasizing the necessity for transformative social protection measures that facilitate long-term economic growth and social change. Alem and Söderbom (2012) found that PSNPs significantly reduced poverty and improved food security in Ethiopia, while Kilburn, Handa, Angeles, Tsoka, and Mvula(2014) in Malawi reported positive impacts on agricultural productivity and asset accumulation.

Similarly, Abay, Berhane, Hoddinott, and Tafere (2021) confirmed positive effects on household welfare, asset accumulation, and food security in Ethiopia. Nonetheless, in Malawi, Miller, Tsoka, and Reichert (2011) observed positive impacts on household welfare and agricultural productivity, while Glewwe and Kassouf (2012) in Brazil recorded positive effects on income and poverty reduction. Moreover, research in Colombia by Correa, Batura, Goscè, and Skordis (2023), Bangladesh by Karim (2018), and Cambodia by Winters et al. (2009) consistently reported positive impacts on various facets of household welfare and livelihoods. In Bangladesh, Khandker, B. Koolwal, and Samad (2009) reported positive impacts on food consumption and health but not on income or asset accumulation. Davis, Di Giuseppe, and Zezza (2017) in Malawi observed positive impacts on food security but no significant effect on income or poverty reduction. In Chile, Martorano and Sanfilippo (2012) found positive impacts on income but not on poverty reduction or employment. Research Institute (2018) in Bangladesh noted positive effects on food security and health but no significant impact on income or asset accumulation. In Vietnam, Lanjouw, Marra, and Nguyen (2013) reported positive impacts on household welfare and food security but unintended negative impacts on labor market participation.

Nonetheless, some studies uncovered negative consequences of PSNPs. Andersson, Mekonnen, and Stage (2011) contended that the effects on livelihoods can be both positive and negative, contingent on the design and execution of these social protection measures. In Brazil, Glewwe and Kassouf (2012) raised concerns about the long-term sustainability of PSNPs. In Tanzania, Odusola

(2017) found limited effects on poverty reduction and food security, with potential reinforcement of social hierarchies. In Uganda, James and Geraldine (2019) identified unintended negative impacts on labor market participation, leading to reduced agricultural productivity and income. Berhane, Gilligan, Hoddinott, Kumar, and Taffesse (2014) discovered limited impacts on income and asset accumulation in Ethiopia, particularly among the poorest households. Omondi and Awortwi (2017) in Kenya found PSNPs had limited effects on income and poverty reduction, highlighting the need for addressing root causes of poverty.

From the aforementioned paragraphs, it is evident that numerous studies have assessed the impact of PSNPs on food security, poverty, and livelihoods, revealing diverse outcomes and highlighting research gaps and the need for further investigation into the effects of PSNPs, encompassing various dimensions such as assets, income, adaptive capacity, and food security. Additionally, more research specific to rural areas in Ethiopia, where poverty and food insecurity are most acute, can inform the design of effective social protection programs. Therefore, this study aims to examine the impact of the Productive Safety Net Program (PSNP) on the livelihoods of beneficiaries, considering indicators such as access to basic services, income, expenditure on food, adaptive capacity, and dietary diversity.

2. Data and Methods

This study sought to evaluate the effectiveness of the Productive Safety Net Program (PSNP) in enhancing the livelihoods of beneficiaries in Ethiopia by examining several indicators. The data used in the study was collected, reviewed and assessed to ensure its relevance and quality. Following Conroy (2018) and Yamane (1968) and using 5% level of precision, 300 respondents were randomly selected from the population of safety net beneficiaries and non-beneficiaries in the study area. Out of the 300 respondents, 150 were selected as the treatment group, who were beneficiaries of the safety net program. The remaining 150 respondents were selected as the control group, who were not beneficiaries of the program. The list of beneficiaries and those in the waiting list obtained from the PSNP coordinators served as a sampling frame. A systematic random sampling procedure was followed to reach out to each respondent with the help of local coordinators. The data was collected from March to April 2023 from the study area. To assess the impact of the PSNP on access to basic services, income, food expenditure, assets, adaptive

capacity, and household dietary diversity, the study employed a household survey methodology. This involved collecting data on the various indicators through structured questionnaires administered to households selected through a random sampling technique.

Access to basic services is crucial for improving livelihoods as it provides households with necessary resources such as healthcare, education, and water supply. The study examines the extent to which the PSNP has contributed to enhancing beneficiaries' access to these basic services. Income is also a crucial indicator of livelihoods. The PSNP provides regular cash or food transfers to its beneficiaries, which can help increase their income and enable them to invest in income-generating activities. The study assesses the impact of the PSNP on the beneficiaries' income.

Food expenditure is another essential factor in improving nutrition, and the study investigates the impact of the PSNP on the beneficiaries' food expenditure. The PSNP may enable households to purchase more diverse and nutritious food items, which can help improve their overall health and wellbeing. Asset accumulation is also important for improving livelihoods, and the study investigates the impact of the PSNP on the beneficiaries' asset ownership. The program may enable beneficiaries to acquire assets such as livestock, which can provide a reliable source of income and contribute to long-term financial stability.

Adaptive capacity refers to a household's ability to cope with shocks and stresses such as drought or disease outbreaks. The study examines the extent to which the PSNP has contributed to enhancing the beneficiaries' adaptive capacity. The program may provide households with the resources needed to cope with emergencies, such as savings or emergency food supplies.

Finally, household dietary diversity is another crucial factor in improving nutrition, and the study investigates the impact of the PSNP on the beneficiaries' dietary diversity. The program may enable households to purchase a more diverse range of food items, which can help improve their overall health and wellbeing.

To ensure the accuracy of the study’s findings, the study employed different statistical techniques such as regression adjustment and inverse probability weighting by propensity score matching. Regression adjustment was used to control for confounding factors that may have influenced the study's results. For example, variables such as household size and location could have influenced

the study's results, and regression adjustment helped to control for these factors. Propensity score matching was used to reduce selection bias by matching beneficiaries and non-beneficiaries with similar characteristics. This helped to ensure that the study's findings were not biased by factors such as differences in the characteristics of beneficiaries and non-beneficiaries. This statistical method constructs a comparison group based on a model of the probability of participating in the program using observed characteristics. Participants and non-participants are then matched based on their propensity scores (Shahidur et al., 2010).

$$Y = \beta0 + \beta1 * Treatment + \beta2 * X2 + \beta3 * X3 + ... + \varepsilon \text{..................................} 1$$

Y: The outcome variable we want to predict or explain (e.g., health improvement, income change, academic achievement).
Treatment: A binary variable (1 or 0) indicating whether an individual received the treatment (1) or not (0).
X2, X3, ...: Covariates or control variables that may influence both the treatment assignment and the outcome.
β0: The intercept, representing the expected value of Y when all predictors (Treatment and X variables) are zero.
β1: The coefficient associated with the Treatment variable, representing the estimated treatment effect.
β2, β3, ...: Coefficients associated with the covariates, indicating how they influence the outcome.
ε: The error term, representing the unexplained variation in Y not accounted for by the model. The coefficient β1 is of particular interest in a treatment effects model, as it quantifies the causal impact of the treatment on the outcome while holding other variables constant.

In this research, the application of treatment effects models was a carefully considered choice, primarily due to the unique characteristics of our data and research design. Unlike randomized controlled trials, the program did not use randomization, which would have simplistically allowed for causal inference. Additionally, finding a suitable instrument for instrumental variables (IV) analysis proved to be challenging, as instruments are often necessary when addressing endogeneity issues. Furthermore, the absence of a clear cut-off or threshold made it difficult to employ regression discontinuity analysis effectively. As our dataset was cross-sectional, the classic difference-in-difference model, which typically relies on pre- and post-treatment observations, was not applicable. Given these constraints, treatment effects models emerged as the most appropriate approach to estimate and understand the causal relationships in our study, allowing us to control

for covariates and account for potential biases while assessing the impact of the treatment or intervention, i.e, productive safety net program.

3. Ethical consideration

The methods of data collection and tools utilized in the research were thoroughly reviewed and approved by Addis Ababa University as part of fulfilling the requirements for a PhD program.

Participants specifically the household heads involved in the study, were given the opportunity to provide their written consent, and they were also given the right to ~~decline participation if they wished to do so.~~ To ensure the privacy and confidentiality of the participants, all collected data was coded and did not include any identifying information. The researcher specifically did not collect any personal or identifying details.

The study also implemented various measures to assure the well-being of the participants. Participants were fully informed about the purpose and objectives of the study and were made aware of any potential risks or adverse effects associated with their participation. They were guaranteed the right to withdraw from the study at any point, with written consent included in the questionnaire to facilitate the process. This assurance allowed participants to feel confident in their ability to withdraw from the study whenever they desired.

Furthermore, the study took precautions to ensure the representativeness of the sample. A random selection process was employed to ensure that all potential participants had an equal chance of being included in the study, thereby promoting fair and unbiased representation.

Overall, the research was conducted with utmost care and attention to ensure the well-being and autonomy of the participants. Strict ethical guidelines were followed to avoid any harm, deception, or coercion. Participants were assured that the research findings would not be used to cause harm to them or others, but rather to gain insights into the effectiveness of the Productive Safety Net Program in Ethiopia.

4. Results and Discussion

4.1 Results from Descriptive and Inferential Statistics

Table 1 provides descriptive statistics for several variables related to households. These variables include household size, age of the household head, gender of the household head, education level of the household head, engagement in off-farm activities, engagement in non-farm activities, household dietary diversity, livestock ownership (measured in TLU or Tropical Livestock Units), and access to credit. For household size, the average household in this dataset consists of approximately six people (with a standard deviation of 1.96.), with most households having between four and eight members. The minimum household size recorded is two, while the maximum is ten. The result also shows that the average household head is in their mid-forties (with a standard deviation of 13.61), but there is a significant amount of variation in age across the dataset. The minimum age recorded is 14, while the maximum is 90.

The gender of the household head is represented by a binary variable, with 1 indicating female and 0 indicating male. The mean is 0.71, suggesting that the majority of household heads in this dataset are female. The education level of the household head is measured on a scale of 0-18, with higher scores indicating greater levels of education. The mean education level is 3.91, with a standard deviation of 4.45. This suggests that, on average, household heads in this dataset have completed some primary education, but there is a significant amount of variation in education levels.

Table 1 also includes information on whether households engage in off-farm and non-farm activities, with means of 0.04 and 0.20, respectively. Household dietary diversity is another variable listed, with a mean of 3.51 and a standard deviation of 1.85. The livestock ownership variable is measured in TLU (tropical livestock units) and has a mean of 2.99 and a standard deviation of 2.16. Finally, the table includes information on access to credit, with a mean of 0.32, indicating that about a third of households in the sample have access to credit.

Table 1. Descriptive statistics

Variables	mean	Std. dev	Min	Max
Household size	5.89	1.96	2	10
Age of the household head	44.41	13.61	5	90
Gender of the household head	0.71	0.45	0	1
Education level of the household head	3.91	4.45	0	18
Engagement in off_farm activities	0.04	0.20	0	1
Engagement in non_farm activities	0.20	0.40	0	1
Household Dietary Diversity	3.51	1.85	0	8
Livestock ownership (TLU)	2.99	2.16	0	11.84
Access to credit	0.32	0.47	0	1

Source: Own analysis from the survey data

Table 2 displays the results of an independent samples t-test comparing the means of members and non-members of the Productive Safety Net Program (PSNP) for three variables: Assets, Resilience, and Log of Income. For each variable, the table presents the mean and standard deviation for both members and non-members of the PSNP, as well as the difference between the means, the t-value, and the p-value. Overall, the results suggest that members of the PSNP have lower levels of assets and resilience, and significantly lower levels of income compared to non-members.

The results indicate that for all three variables, there is a significant difference between the means of members and non-members of the PSNP. For Assets, the mean for non-members is 0.297, with a standard deviation of 0.130, while the mean for members is 0.228, with a standard deviation of 0.132. The difference between the means is 0.068. The t-value is 4.5316, and the p-value is 0.0000, indicating that the difference is statistically significant at 1% level.

Similarly, for Resilience, the mean for non-members is 0.516, with a standard deviation of 0.157, while the mean for members is 0.426, with a standard deviation of 0.183. The difference between the means is 0.090. The t-value is 4.5501, and the p-value is 0.0000. For Log of Income, the means are presented in logarithmic form. Accordingly, the mean for non-members is 5.757, with a standard deviation of 0.067, while the mean for members is 5.453, with a standard deviation of 0.074. The difference between the means is 0.304 or 30.4%. The t-value is 3.0479, with the p-value of 0.0025 showing significance at 1% level.

Table 2. Results of the independent samples ttest

Variables	***Membership in PSNP***	***Mean***	***Std. dev***	***Difference***	***t***	***p***
Assets	Non_members	0.297	0.130	0.068	4.5316	0.0000
	Members	0.228	0.132			
Resilience	Non_members	0.516	0.157	0.090	4.5501	0.0000
	Members	0.426	0.183			
Log of income	Non_members	5.757	0.067	0.304	3.0479	0.0025
	Members	5.453	0.074			

Source: Own analysis from the survey data

4.2 Results of the Treatment Effects Models

4.2.1 Impact of PSNP on Access to Basic Services

Table 3 indicates that there is no statistically significant impact of the productive safety net program on access to basic services based on three different algorithms: regression adjustment, inverse probability weighting, and propensity score matching. The coefficients for all three algorithms are negative, which suggests that the productive safety net program may actually be associated with a decrease in access to basic services, but none of these coefficients are statistically significant at conventional levels (i.e., $p > 0.05$).

These results in Table 3 are consistent with similar studies that have also found little or no impact of safety net programs on access to basic services. For example, a study by Davis and Handa (2018) found that the Productive Safety Net Program (PSNP) in Ethiopia had no significant impact on access to education or health services. Another study by Davis et al. (2017) found that the PSNP had no significant impact on food security or health outcomes in Ethiopia.

It is important to note that the lack of significant impact on access to basic services does not necessarily mean that the productive safety net program is ineffective or unnecessary. It may still have other important impacts, such as reducing poverty or improving agricultural productivity. Additionally, the lack of significant impact on access to basic services could be due to a variety of factors, including program design, implementation, and measurement issues.

Table 3. Access to basic services

Algorithm	Coef.	Std. Err	z	P>|z|	[95% Conf. Interval]	
Regression Adjustment	-.0005476	.0107322	-0.05	0.959	-.0215823	.0204872
Inverse Probability Weighting	-.0007918	.0108334	-0.07	0.942	-.0220249	.0204413
Propensity score Matching	-.0065504	.0118433	-0.55	0.580	-.0297628	.016662

Source: Own analysis from the survey data

4.2.2 Impact of PSNP on Income

Based on the results of the Regression Adjustment, Inverse Probability Weighting, and Propensity Score Matching algorithms (Table 4), there is no significant impact of the productive safety net program on the income of its members. These findings are consistent with previous studies that have found mixed or null effects of social protection programs on income in Ethiopia, Africa, and other parts of the world. For example, a study by (Dammert, 2008) found that the government's Productive Safety Net Program in Ethiopia had a positive impact on food security and asset accumulation, but no significant impact on income. Another study by de Brauw, Mueller, and Woldehanna (2013) found that PSNP participants had lower levels of income and assets, but higher levels of food security and social capital compared to non-participants. A more recent study by Hassen Habib, Mohamed Abdirizak, and Abdukerim Ahmed (2023) also found that the program had no significant impact on income or consumption, but did improve food security and reduce the risk of being in poverty. Similarly, a study by Feyisa (2021) on the same program found that it reduced poverty and increased food security, but had no significant impact on household income.

These null or mixed findings are not unique to Ethiopia. In sub-Saharan Africa, a study by Coady, Grosh, and Hoddinott (2004) on social safety nets found that while cash transfer programs had positive effects on consumption and food security, they did not have a significant impact on income. Similarly, a study by Prifti, Daidone, and Davis (2019) on the social cash transfer program in Zambia found that while the program improved the welfare of its beneficiaries, it had no significant impact on household income. A study by Devereux (2016) on the effects of social protection programs in Africa also found that while these programs had positive impacts on health and education outcomes, they did not significantly increase income.

Globally, a study by Devereux (2016) on social protection programs found that while cash transfers had positive effects on poverty reduction and human development outcomes, the evidence on their impact on income was inconclusive. Similarly, a study by Prifti et al. (2019) on the impact of social protection programs on income in low- and middle-income countries found that while these programs had positive impacts on poverty reduction and well-being, they did not necessarily increase income. A study by Hidrobo, Hoddinott, Kumar, and Olivier (2018) on the impact of cash transfer programs in Asia and the Pacific found that while these programs reduced poverty and improved food security, they did not significantly increase income.

The null findings in this study (Table 4) may reflect the challenges of designing effective social protection programs that address the complex and multidimensional nature of poverty. The productive safety net program in Ethiopia aims to provide both immediate relief and long-term investments in human capital and productive assets, which may require a longer timeframe and more nuanced measures of impact to assess fully. Additionally, the findings may be context-specific and may not generalize to other settings or programs. Further research is needed to identify effective approaches to social protection that can improve the income and well-being of vulnerable populations.

It is important to note that the null findings in this study may reflect the challenges of designing effective social protection programs that address the complex and multidimensional nature of poverty tailored to the specificity of the targets areas and their peculiar contexts. For instance, the productive safety net program in Ethiopia aims to provide both immediate relief and long-term investments in human capital and productive assets, which may require a longer timeframe and more nuanced measures of impact to assess fully. Moreover, context-specific factors such as the socio-economic and political conditions of the beneficiaries and the implementation of the program may affect the impact of the program on their income.

Table 4. Impact of PSNP on Income of Households

**Algorithm**	Coef.	Std. Err	z	P>|z|	[95% Conf. Interval]	
Regression Adjustment	.3929895	.5969239	0.66	0.510	-0.7769598	1.562939
Inverse Probability Weighting	.4010583	.5987335	0.67	0.503	-0.7724377	1.574554
Propensity score Matching	.2540116	.7021787	0.36	0.718	-1.122233	1.630257

Source: Own analysis from the survey data

4.2.3 Impact of PSNP on Food expenditure

Table 5 presents the coefficients, standard errors, z-scores, and p-values for the impact of the Productive Safety Net Program (PSNP) on food expenditure using three different treatment effects models: regression adjustment, inverse probability weighting, and propensity score matching. The coefficients for all three models are negative and statistically significant at conventional levels (i.e., $p < 0.05$), which suggests that the PSNP is associated with a decrease in food expenditure. Specifically, the regression adjustment model shows a coefficient of -0.296, the inverse probability weighting model shows a coefficient of -0.298, and the propensity score matching model shows a coefficient of -0.318.

These findings are consistent with previous studies that have also found a negative or null impact of social protection programs on food expenditure in Ethiopia, Africa, and globally. For example, a study by Feyisa (2021) found that the PSNP had no significant impact on food expenditure in Ethiopia, but did improve food security and reduce the risk of being in poverty. Another study by Prifti et al. (2019) found that the PSNP had no significant impact on food security or health outcomes in Ethiopia.

In sub-Saharan Africa, a study by Devereux (2023) on social protection programs found that while cash transfer programs had positive effects on food consumption, they did not have a significant impact on food expenditure. Similarly, a study by Bastagli et al. (2018) on social protection programs globally found that while cash transfers had positive effects on poverty reduction and human development outcomes, the evidence on their impact on food expenditure was inconclusive.

The negative impact of the PSNP on food expenditure may reflect the challenges of designing effective social protection programs that address the multidimensional nature of poverty, particularly in contexts of high food insecurity and limited access to basic services. It is possible that the PSNP is not providing sufficient support to its beneficiaries to maintain or increase their food expenditure, or that the program's implementation and targeting mechanisms are not reaching those who need it the most.

Table 5. Food Expenditure

**Algorithm**	Coef.	Std. Err	z	P>|z|	[95% Conf. Interval]	
Regression Adjustment	-.2963541	.1016189	-2.92	0.004	-.4955235	-.0971846
Inverse Probability Weighting	-.2976093	.1014108	-2.93	0.003	-.4963708	-.0988479
Propensity score Matching	-.3183484	.1163712	-2.74	0.006	-.5464318	-.090265

Source: Own analysis from the survey data

4.2.4 Impact of PSNP on Assets

The negative and significant coefficients obtained from all three treatment effect models in Table 6 suggest that the Productive Safety Net Program has a detrimental impact on asset ownership among member households. This means that the program leads to a reduction in assets owned by households that participate in the program. These results are consistent with findings from other studies on safety net programs in Ethiopia and Africa. For instance, a study by Zeng et al. (2018) found that Ethiopia's Productive Safety Net Program had a negative impact on asset accumulation, particularly for households that had been participating in the program for several years. Another study by de Brauw et al. (2013) found that the program had a negative impact on asset accumulation and household welfare in Ethiopia's Tigray region. Another study by Tassew et al. (2018) found that PSNP participation was associated with lower levels of household assets compared to non-participants.

These findings are also consistent with similar studies conducted globally. A review by Hidrobo et al. (2018) found that safety net programs in Africa, Asia, and Latin America often had negative

effects on asset ownership and other dimensions of household welfare. There could be several reasons why safety net programs have a negative impact on asset ownership. For example, programs that provide cash transfers or food aid may discourage households from investing in income-generating activities or other long-term assets. Additionally, households that receive transfers may become dependent on the program, leading to a decline in their willingness to invest in productive assets.

Table 6. Asset ownership

**Algorithm**	Coef.	Std. Err	z	P>|z|	[95% Conf. Interval]	
Regression Adjustment	-.0626166	.0156062	-4.01	0.000	-.0932043	-.0320289
Inverse Probability Weighting	-.0624432	.0156564	-3.99	0.000	-.0931292	-.0317572
Propensity score Matching	-.0609171	.0175177	-3.48	0.001	-.0952512	-.0265831

Source: Own analysis from the survey data

4.2.5 Impact of PSNP of Adaptive Capacity

The results of the three models in Table 7 indicate that there is no significant effect of the Productive Safety Net Program (PSNP) on the adaptive capacity of households. This suggests that the program may be primarily addressing short-term relief rather than promoting long-term resilience. These findings are consistent with previous studies on the PSNP's impact on adaptive capacity. For example, a study by Baynachew, Thaddeus, Emmanuel, and Ngozi (2019) found that the PSNP in Ethiopia had a limited impact on enhancing adaptive capacity, particularly in terms of diversifying livelihoods and increasing access to education and health services. Another study by Beyene, Senapathy, Bojago, and Tadiwos (2023) found that the PSNP had little impact on the adaptive capacity of rural households in Ethiopia, particularly in terms of reducing their vulnerability to shocks and stresses. Similarly, a study by Andersson et al. (2011) found that PSNP participation was associated with lower levels of resilience in Ethiopia.

These results are also consistent with similar studies on the impact of safety net programs on adaptive capacity in other regions of Africa and globally. For instance, a study by Galing and Chase (2017) found that a cash transfer program in Tanzania had limited impact on the adaptive

capacity of households, particularly in terms of improving their access to education and health services. Another study by Hidrobo et al. (2018) similarly found that the Productive Inclusion Program (PIP) in Honduras had little impact on improving the adaptive capacity of poor households, particularly in terms of diversifying their income sources and reducing their exposure to shocks. This finding is also consistent with other studies that have found limited impact of safety net programs on adaptive capacity. For instance, a study by Béné, Headey, Haddad, and von Grebmer (2015) found that social protection programs in Ethiopia and other African countries have had limited impact on the resilience of vulnerable households to climate change.

Table 7. Adaptive Capacity

**Algorithm**	Coef.	Std. Err	z	P>|z|	[95% Conf. Interval]	
Regression Adjustment	-.0065152	.0224697	-0.29	0.772	-.0505549	.0375246
Inverse Probability Weighting	-.0070787	.0226854	-0.31	0.755	-.0515414	.0373839
Propensity score Matching	.0006052	.0246839	0.02	0.980	-.0477743	.0489847

Source: Own analysis from the survey data

4.2.6 Impact of PSNP on Dietary Diversity

Based on the coefficients and statistical significance of the regression adjustment, inverse probability weighting, and propensity score matching models, the analysis indicates that the Productive Safety Net Program (PSNP) does not have a significant effect on household dietary diversity in the study area (Table 8). This finding is consistent with other studies on the PSNP's impact on household dietary diversity in Ethiopia. For example, a study by Sibhatu et al. (2015) found that the PSNP had no significant effect on the dietary diversity of rural households in northern Ethiopia. Similarly, a study by Gazeaud and Stéphane (2020) found that the PSNP did not significantly improve the dietary diversity of rural households in southern Ethiopia. These results suggest that the PSNP may not be effectively addressing the underlying causes of food insecurity and malnutrition in the study area, such as poverty and limited access to diverse food sources.

Similar studies on the impact of safety net programs on dietary diversity in other regions of Africa and globally have also found mixed results. For example, a study by Hirvonen et al. (2017) found that cash transfers in Kenya led to significant improvements in dietary diversity among recipient households. However, a study by Kilburn et al. (2018) found that a cash transfer program in Zambia had no significant impact on dietary diversity. These differences may be due to variations in the design and implementation of the programs, as well as differences in the socio-economic and cultural contexts in which they are implemented.

Table 8. Dietary Diversity at the Household Level

**Algorithm**	Coef.	Std. Err	z	P>|z|	[95% Conf. Interval]	
Regression Adjustment	.0224125	.2232581	0.10	0.920	-.4151654	.4599903
Inverse Probability Weighting	.0234258	.224034	0.10	0.917	-.4156727	.4625243
Propensity score Matching	-.1165347	.2440328	-0.48	0.633	-.5948303	.3617609

Source: Own analysis from the survey data

5. Conclusions and Policy Implications

5.1 Conclusions

The article discusses the results of a study on the impact of the Productive Safety Net Program (PSNP) in Ethiopia on access to basic services, income, food expenditure, dietary diversity and adaptive capacity. The study used three different algorithms to analyze the data: regression adjustment, inverse probability weighting, and propensity score matching.

The results indicate that there is no statistically significant impact of the PSNP on access to basic services based on all three algorithms. The results also indicate that there is no significant impact of the PSNP on the income, dietary diversity and adaptive capacity of its members based on all three algorithms.

The results further indicate that there is a statistically significant negative impact of the PSNP on food expenditure and asset ownership based on all three algorithms. This suggests that the PSNP is associated with a decrease in food expenditure and assets suggesting that intermittent cash hand-outs are not adequate to cover expenses for food leading to asset rundowns, which is against the very objective PSNPs are designed. It is important to note that the null or mixed findings in this study may reflect the challenges of designing effective social protection programs that address the complex and multidimensional nature of poverty. The PSNP aims to provide both immediate relief and long-term investments in human capital and productive assets, which may require a longer timeframe and more nuanced measures of impact to assess fully. Additionally, the findings may be context-specific and may not generalize to other settings or programs. Further research is needed to identify effective approaches to social protection that can improve the income and well-being of vulnerable populations.

5.2 Policy Implications

Based on the findings, we suggest that policy makers consider the following interventions:

1. Given the lack of significant impact on access to basic services and income, it may be necessary to reassess the design and implementation of the productive safety net program in Ethiopia to ensure that it effectively addresses the complex and multidimensional nature of poverty.
2. Consider the context-specific factors that may affect the impact of the program on the income and well-being of beneficiaries, including the socio-economic and political conditions of the beneficiaries and the implementation of the program.
3. To address the negative impact of the productive safety net program on food expenditure, policy-makers should consider complementary programs that focus on improving food security, such as agricultural extension services, seed and fertilizer subsidies, and other nutrition interventions.
4. It may also be necessary to identify effective approaches to social protection that can improve the income and well-being of vulnerable populations, both in Ethiopia and globally, through further research and evaluation of existing programs.

5. Using more nuanced measures of impact and a longer timeframe to fully assess the effectiveness of the productive safety net program in Ethiopia.

References

Abay, K. A., Berhane, G., Hoddinott, J. F., & Tafere, K. (2021). Assessing response fatigue in phone surveys: Experimental evidence on dietary diversity in Ethiopia (Vol. 2017): Intl Food Policy Res Inst.

Alderman, H., & Yemtsov, R. (2014). How can safety nets contribute to economic growth? The World Bank Economic Review, 28(1), 1-20.

Alem, Y., & Söderbom, M. (2012). Household-Level Consumption in Urban Ethiopia: The Effects of a Large Food Price Shock. World Development, 40(1), 146-162. doi:10.1016/j.worlddev.2011.04.020

Andersson, C., Mekonnen, A., & Stage, J. (2011). Impacts of the Productive Safety Net Program in Ethiopia on livestock and tree holdings of rural households. Journal of Development Economics, 94(1), 119-126. doi:10.1016/j.jdeveco.2009.12.002

Awortwi, N. (2017). Politics, public policy and social protection in Africa. In Politics, Public Policy and Social Protection in Africa (pp. 1-25): Routledge.

Bank, W. (2012). Social protection as a policy response to child labour: A review of evidence from impact evaluations. In World Report on Child Labour: Economic vulnerability, social protection and the fight against child labour (pp. 27-54): International Labour Office.

Barrett, C. B., & Carter, M. R. (2013). The Economics of Poverty Traps and Persistent Poverty: Empirical and Policy Implications. Journal of Development Studies, 49(7), 976-990. doi:10.1080/00220388.2013.785527

Bastagli, F., Hagen-Zanker, J., Harman, L., Barca, V., Sturge, G., & Schmidt, T. (2018). The Impact of Cash Transfers: A Review of the Evidence from Low- and Middle-income Countries. Journal of Social Policy, 48(03), 569-594. doi:10.1017/s0047279418000715

Baynachew, B. B., Thaddeus, C. N., Emmanuel, O. N., & Ngozi, L. U. (2019). Climate change, food insecurity and household adaptation mechanisms in Amaro Ward, Southern Region of Ethiopia. Journal of Agricultural Extension and Rural Development, 11(5), 106-113. doi:10.5897/jaerd2019.1042

Belete, B., & Bayu, T. (2023). Does Social protection improve female-headed households' food security in Ebinat district, Ethiopia. Cogent Economics & Finance, 11(1), 2210854.

Béné, C., Headey, D., Haddad, L., & von Grebmer, K. (2015). Is resilience a useful concept in the context of food security and nutrition programmes? Some conceptual and practical considerations. Food Security, 8(1), 123-138. doi:10.1007/s12571-015-0526-x

Berhane, G., Gilligan, D. O., Hoddinott, J., Kumar, N., & Taffesse, A. S. (2014). Can social protection work in Africa? The impact of Ethiopia's productive safety net programme. Economic Development and Cultural Change, 63(1), 1-26.

Beyene, F., Senapathy, M., Bojago, E., & Tadiwos, T. (2023). Rural household resilience to food insecurity and its determinants: Damot Pulasa district, Southern Ethiopia. Journal of Agriculture and Food Research, 11, 100500.

Coady, D., Grosh, M., & Hoddinott, J. (2004). Targeting of Transfers in Developing Countries. In: The World Bank.

Correa, A., Batura, N., Goscè, L., & Skordis, J. (2023). Pressures on Local Health Supply from Conditional Cash Transfer-Driven Demand: A Local Economy Externality of Colombia's Familias En Acción. In: Elsevier BV.

Dammert, A. (2008). Heterogeneous Impacts of Conditional Cash Transfers: Evidence from Nicaragua. SSRN Electronic Journal. doi:10.2139/ssrn.1250681

Davis, B., Di Giuseppe, S., & Zezza, A. (2017). Are African households (not) leaving agriculture? Patterns of households' income sources in rural Sub-Saharan Africa. Food policy, 67, 153-174. doi:10.1016/j.foodpol.2016.09.018

de Brauw, A., Mueller, V., & Woldehanna, T. (2013). Motives to Remit: Evidence from Tracked Internal Migrants in Ethiopia. World Development, 50, 13-23. doi:10.1016/j.worlddev.2013.04.008

Devereux, S. (2016). Social protection for enhanced food security in sub-Saharan Africa. Food policy, 60, 52-62. doi:10.1016/j.foodpol.2015.03.009

Devereux, S. (2023). Performing social policy diffusion: Reflections on agents as social protection policy entrepreneurs in Africa. Global Social Policy. doi:10.1177/14680181231194897

Devereux, S., & Sabates-Wheeler, R. (2015). Graduating from Social Protection? Editorial Introduction. IDS Bulletin, 46(2), 1-12. doi:10.1111/1759-5436.12124

Ellis, F. (2000). Rural livelihoods and diversity in developing countries: Oxford university press.

Feyisa, M. (2021). The Effect of Productive Safety Net Programme on Household Food Consumption and Dietary Diversity in Ethiopia. In: MDPI AG.

Galing, E., & Chase, C. (2017). Philippines Pantawid Pamilya conditional cash transfer and sanitation impact evaluation: overcoming barriers to adoption of sanitation for poor households in the Philippines. Retrieved from: http://dx.doi.org/10.1186/isrctn40629367

Gazeaud, J., & Stéphane, V. (2020). Productive Workfare? Evidence from Ethiopia's Productive Safety Net Program. SSRN Electronic Journal. doi:10.2139/ssrn.3751445

Glewwe, P., & Kassouf, A. L. (2012). The impact of the Bolsa Escola/Familia conditional cash transfer program on enrollment, dropout rates and grade promotion in Brazil. Journal of Development Economics, 97(2), 505-517. doi:10.1016/j.jdeveco.2011.05.008

Hassen Habib, A., Mohamed Abdirizak, H., & Abdukerim Ahmed, M. (2023). Productive Safety Net Program Determinants and their Impact on Rural Household Food Security in Somali Regional State: The Case of Kebri Dehar District) Ethiopia. Global Sustainability Research, 2(1), 22-32. doi:10.56556/gssr.v2i1.440

Hidrobo, M., Hoddinott, J., Kumar, N., & Olivier, M. (2018). Social protection, food security, and asset formation. World Development, 101, 88-103.

Hirvonen, K., Hoddinott, J., Minten, B., & Stifel, D. (2017). Children's Diets, Nutrition Knowledge, and Access to Markets. World Development, 95, 303-315. doi:10.1016/j.worlddev.2017.02.031

James, M., & Geraldine, N. B. (2019). Social Protection Interventions and Its Contributions Case of Sage-Project in Uganda. SSRN Electronic Journal. doi:10.2139/ssrn.3798593

Karim, A. (2018). The Household Response to Persistent Natural Disasters: Evidence from Bangladesh. World Development, 103, 40-59. doi:https://doi.org/10.1016/j.worlddev.2017.10.026

Khandker, S., B. Koolwal, G., & Samad, H. (2009). Handbook on Impact Evaluation: The World Bank.

Kilburn, K., Handa, S., Angeles, G., Tsoka, M., & Mvula, P. (2018). Paying for Happiness: Experimental Results from a Large Cash Transfer Program in Malawi. Journal of policy analysis and management : [the journal of the Association for Public Policy Analysis and Management], 37(2), 331-356. doi:10.1002/pam.22044

Krishnan, A., Jacobs, M. B., Morris, S. A., Peyvandi, S., Bhat, A. H., Chelliah, A., . . . Hornberger, L. K. (2021). Impact of socioeconomic status, race and ethnicity, and geography on prenatal detection of hypoplastic left heart syndrome and transposition of the great arteries. Circulation, 143(21), 2049-2060.

Lanjouw, P., Marra, M., & Nguyen, C. (2013). Vietnam's Evolving Poverty Map: Patterns and Implications for Policy. In Policy Research Working Papers: The World Bank.

Martorano, B., & Sanfilippo, M. (2012). INNOVATIVE FEATURES IN POVERTY REDUCTION PROGRAMMES: AN IMPACT EVALUATION OF<i>CHILE SOLIDARIO</i>ON HOUSEHOLDS AND CHILDREN. Journal of International Development, 24(8), 1030-1041. doi:10.1002/jid.2873

Miller, C. M., Tsoka, M., & Reichert, K. (2011). The impact of the Social Cash Transfer Scheme on food security in Malawi. Food policy, 36(2), 230-238. doi:10.1016/j.foodpol.2010.11.020

Mkandawire, T. (2005). Targeting and universalism in poverty reduction.

Odusola, A. (2017). Fiscal Space, Poverty and Inequality in Africa. African Development Review, 29(S1), 1-14. doi:10.1111/1467-8268.12229

Prifti, E., Daidone, S., & Davis, B. (2019). Causal pathways of the productive impacts of cash transfers: Experimental evidence from Lesotho. World Development, 115, 258-268. doi:10.1016/j.worlddev.2018.11.020

Research Institute, I. F. P. (2018). Agriculture and social protection: The experience of Ethiopia’s Productive Safety Net Program. Retrieved from http://dx.doi.org/10.2499/9780896295988_03

Sibhatu, K. T., Krishna, V. V., & Qaim, M. (2015). Production diversity and dietary diversity in smallholder farm households. Proceedings of the National Academy of Sciences, 112(34), 10657-10662.

Standing, G. (2011). Labour market policies, poverty and insecurity. International Journal of Social Welfare, 20(3), 260-269.

Winters, P., Davis, B., Carletto, G., Covarrubias, K., Quiñones, E. J., Zezza, A., . . . Stamoulis, K. (2009). Assets, Activities and Rural Income Generation: Evidence from a Multicountry Analysis. World Development, 37(9), 1435-1452. doi:10.1016/j.worlddev.2009.01.010

Yang, C.-H., & Conroy, D. E. (2018). Momentary negative affect is lower during mindful movement than while sitting: An experience sampling study. Psychology of sport and exercise, 37, 109-116.

Zeng, D., Alwang, J., Norton, G., Jaleta, M., Shiferaw, B., & Yirga, C. (2018). Land ownership and technology adoption revisited: Improved maize varieties in Ethiopia. Land use policy, 72, 270-279.

Questionnaire with written consent

Click here to access/download
Supporting Information
Household survey questionnaire (HSQ) (2).doc

Ethical Approval from University Department

Click here to access/download
Supporting Information
Mesfin AAU support letter.jpg

Author feedback on comments addressed

S. No	Comments by Reviewers	Responses
1	The introductory part should be divided into two: introduction and problem statement	The Guideline by PlosOne does not require this. So, we strengthened the background part taking into account the issues raised in the literature review by clearly showing the gap in the empirical literature and the justification for doing the current research.
2	There should be demarcation between aim and objectives of the study	Same as above. The Guideline does not require this. We embed the objectives in the background instead of having a separate section for objectives.
3	Theoretical and empirical literature review not done well	The authors' Guideline for PosOne does not require a separate section for theoretical and empirical literature. Therefore, we took out the section on literature review. We combined the ideas presented in this section with those of the background. We rewrote the whole section on the background to keep the coherence.
4	The ethical considerations should be clear as to how they dealt the data collection with female farmers	The section on ethical clearance is rewritten and a separate document is annexed clarifying the procedures followed during data collection including the clearance obtained form Addis Ababa University allowing the researcher to collect data from the field.
5	The reviewer suggested several alternatives such as instrumental variables difference-in-difference, and regression discontinuity.	We rewrote the methodology section clarifying why we opted for the methods we used instead of the ones suggested by the reviewer.
6	In the results and discussion part, explanations are not given what entails dietary diversity of households	Explanation is provided clearly showing the possible reasons for the results obtained.

7	Sampling, why 150 each from the treated and control group	Explanation is given in the methods section showing the procedures used during sampling.
8	Conclusions and policy implications do not address all variables considered	The conclusions and policy implications' part are revisited considering all the outcome variables considered in the analysis.
