# Supplementary material for: Does productive safety net program enhance livelihoods? Insights from vulnerable households in Wolaita zone, Ethiopia
Source: PLoS One. 2024 Apr 4;19(4):e0297780. doi: 10.1371/journal.pone.0297780 (PMC10994340; doi:10.1371/journal.pone.0297780)
Supplement: Attachment — Submitted filename: Author Table for Feedback.docx [file pone.0297780.s003.docx]

Author feedback on comments addressed

| S. No | Comments by Reviewers | Responses |
| --- | --- | --- |
| 1 | The introductory part should be divided into two: introduction and problem statement | The Guideline by PlosOne does not require this. So, we strengthened the background part taking into account the issues raised in the literature review by clearly showing the gap in the empirical literature and the justification for doing the current research. |
| 2 | There should be demarcation between aim and objectives of the study | Same as above. The Guideline does not require this. We embed the objectives in the background instead of having a separate section for objectives. |
| 3 | Theoretical and empirical literature review not done well | The authors’ Guideline for PosOne does not require a separate section for theoretical and empirical literature. Therefore, we took out the section on literature review. We combined the ideas presented in this section with those of the background. We rewrote the whole section on the background to keep the coherence. |
| 4 | The ethical considerations should be clear as to how they dealt the data collection with female farmers | The section on ethical clearance is rewritten and a separate document is annexed clarifying the procedures followed during data collection including the clearance obtained form Addis Ababa University allowing the researcher to collect data from the field. |
| 5 | The reviewer suggested several alternatives such as instrumental variables difference-in-difference, and regression discontinuity. | We rewrote the methodology section clarifying why we opted for the methods we used instead of the ones suggested by the reviewer. |
| 6 | In the results and discussion part, explanations are not given what entails dietary diversity of households | Explanation is provided clearly showing the possible reasons for the results obtained. |
| 7 | Sampling, why 150 each from the treated and control group | Explanation is given in the methods section showing the procedures used during sampling. |
| 8 | Conclusions and policy implications do not address all variables considered | The conclusions and policy implications’ part are revisited considering all the outcome variables considered in the analysis. |
