# Supplementary material for: Does productive safety net program enhance livelihoods? Insights from vulnerable households in Wolaita zone, Ethiopia
Source: PLoS One. 2024 Apr 4;19(4):e0297780. doi: 10.1371/journal.pone.0297780 (PMC10994340; doi:10.1371/journal.pone.0297780)
Supplement: Attachment — Submitted filename: Rebuttal JAN 10.docx [file pone.0297780.s005.docx]

Rebuttal

| S. No | Section | Comments by Reviewers | Responses |
| --- | --- | --- | --- |
| 1 | Abstract | Highlight the study design and the software utilized | Done and included. |
| 2 | Abstract: key words | Include variables of the study and remove Impact | Done and included |
| 3 | Background | Focus on each variable to each paragraph | Done and rearranged |
| 4 | Data and methods | Adress same comment from abstract | Done and addressed |
| 5 | Data and methods | Leave sentence which is unnecessary and lack specific | Done and removed |
| 6 | Data and methods | Mention study area by its name | Changed and addressed |
| 7 | Data and methods | Study variable could be presented as definition and question used to describe the variables , categories and codes, frequencies and percentages. | Done and included |
| 8 | Data and methods | Provide the name of the formula in equation | Done and inserted |
| 9 | Data and methods | Add as Appendix | addressed |
| 10 | Ethical consideration | State whether you received ethics approval or waiver approval letter | Addressed, submitted and already got clearance form the journal |
| 11 | Ethical consideration | Omission of repeated statement | Addressed |
| 12 | Ethical consideration | State the measures to assure wellbeing of participants | Addressed and included |
| 13 | Results and discussion | Include the table 1 on methodology rather in results | Addressed and moved |
| 14 | Results and discussion | Put the table 2 before the analysis. | Addressed and moved |
| 15 | Results and discussion | Create on table for all variables in one and include the analysis below. | Addressed in one table and analyses followed |
